# Age-group differences in speech identification despite matched audiometrically normal hearing: contributions from auditory temporal processing and cognition

**DOI:** 10.3389/fnagi.2014.00347

**Published:** 2015-01-13

**Authors:** Christian Füllgrabe, Brian C. J. Moore, Michael A. Stone

**Affiliations:** ^1^MRC Institute of Hearing ResearchNottingham, UK; ^2^Department of Psychology, University of CambridgeCambridge, UK; ^3^School of Psychological Sciences, University of ManchesterManchester, UK; ^4^Central Manchester NHS Hospitals Foundation TrustManchester, UK

**Keywords:** aging, normal hearing, speech identification, temporal envelope, temporal fine structure, cognition

## Abstract

Hearing loss with increasing age adversely affects the ability to understand speech, an effect that results partly from reduced audibility. The aims of this study were to establish whether aging reduces speech intelligibility for listeners with normal audiograms, and, if so, to assess the relative contributions of auditory temporal and cognitive processing. Twenty-one older normal-hearing (ONH; 60–79 years) participants with bilateral audiometric thresholds ≤ 20 dB HL at 0.125–6 kHz were matched to nine young (YNH; 18–27 years) participants in terms of mean audiograms, years of education, and performance IQ. Measures included: (1) identification of consonants in quiet and in noise that was unmodulated or modulated at 5 or 80 Hz; (2) identification of sentences in quiet and in co-located or spatially separated two-talker babble; (3) detection of modulation of the temporal envelope (TE) at frequencies 5–180 Hz; (4) monaural and binaural sensitivity to temporal fine structure (TFS); (5) various cognitive tests. Speech identification was worse for ONH than YNH participants in all types of background. This deficit was not reflected in self-ratings of hearing ability. Modulation masking release (the improvement in speech identification obtained by amplitude modulating a noise background) and spatial masking release (the benefit obtained from spatially separating masker and target speech) were not affected by age. Sensitivity to TE and TFS was lower for ONH than YNH participants, and was correlated positively with speech-in-noise (SiN) identification. Many cognitive abilities were lower for ONH than YNH participants, and generally were correlated positively with SiN identification scores. The best predictors of the intelligibility of SiN were composite measures of cognition and TFS sensitivity. These results suggest that declines in speech perception in older persons are partly caused by cognitive and perceptual changes separate from age-related changes in audiometric sensitivity.

## Introduction

Aging in adults is associated with deterioration and increased effortfulness of all levels of speech processing (from identification to comprehension), especially in noisy and reverberant conditions (e.g., CHABA, 1988). It has long been known that hearing sensitivity declines with increasing age (Bunch, 1929; Corso, 1963) and this is associated with poorer speech identification (Harris et al., 1956; Delk et al., 1957). More recently, it has also become apparent that hearing-impaired people report a lower quality of life (Dalton et al., 2003), experience more social isolation (Weinstein and Ventry, 1982; Strawbridge et al., 2000) and depression (Gopinath et al., 2009; Huang et al., 2010), and show poorer cognitive functioning and accelerated cognitive decline (Lin et al., 2011, 2013) than normal-hearing people. This suggests that speech communication difficulties not only constitute a socio-psychological handicap for the affected person (Arlinger, 2003) but also represent an important financial burden for society in terms of social and health care provision (Mohr et al., 2000; Hjalte et al., 2012; Foley et al., 2014).

Modern digital hearing aids, which provide frequency-specific amplification, at least partially restore audibility of those sounds that would not otherwise be perceived by the hearing-impaired person. These aids are the standard treatment in most cases of hearing loss. While aided speech identification in quiet and background noise generally improves with increasing audibility (e.g., Humes, 2002), the observed benefit often falls short of what would be expected based on audibility (Humes and Dubno, 2010). One possible explanation for this is that age-related changes in supra-threshold auditory processing and cognition—that are not captured by an audiometric assessment—contribute to the speech-identification difficulties of older people (e.g., Humes et al., 2013b; Moore et al., 2014; Schoof and Rosen, 2014).

To study the existence of age effects unrelated to audibility, most previous research adopted a cross-sectional design, in which a group of older participants (generally somewhat arbitrarily taken as ≥ 60 years) was compared to a group of young controls. Given the high prevalence of hearing loss in the older population (Davis, 1995; Cruickshanks et al., 1998; Agrawal et al., 2008), establishing audiometric equality between these age groups to control for the effect of audibility is not easy. Alternative solutions have been sought to matching the age groups, for example by: (1) spectrally shaping the speech signal to equate audibility across groups; (2) distorting the speech signals delivered to the young normal-hearing (YNH) participants (e.g., by adding noise) to simulate the hearing loss of the older participants; or (3) statistically partialling out the effect of hearing loss. None of these approaches controls for possible “central effects of peripheral pathology” (Willott, 1996) in the older participants, i.e., physiological and anatomical changes in the central auditory system induced by peripheral pathology (Robertson and Irvine, 1989; Ison et al., 2010). The approach using mathematical adjustments has the additional disadvantage that, since age and audiometric thresholds are not statistically independent, partialling out the effect of hearing sensitivity also removes some of the age effect, resulting in an underestimation of the effect of the latter (Martin et al., 1991). In the present study, the older participants were selected to have hearing sensitivity matching that of a YNH control group over a wide frequency range and in both ears. In addition, a relatively large number of older normal-hearing (ONH) participants was recruited, to allow calculation of correlations across measures within the ONH group.

Many previous studies of aging focussed on either perceptual *or* cognitive processes involved in speech processing, frequently employing a single measure of the process under study. Here, we attempted to study the interplay and relative contribution of both of these processes in the case of speech-in-noise (SiN) identification, using multiple indices of perceptual, cognitive, and speech processing.

The choice of perceptual tasks was motivated by our knowledge of how sounds are represented or “coded” in the auditory system. Acoustic broadband signals, such as speech, are decomposed in the cochlea into a series of bandpass-filtered signals, each corresponding to a specific position on the basilar membrane. The response at each place can be considered as a temporal envelope (TE; corresponding to the slow variations in overall amplitude over time) imposed on a time-varying carrier, the temporal fine structure (TFS; faster variations corresponding to the rapid oscillations in the filtered waveform). Both types of temporal information are represented in the auditory system by the timing of neural discharges (phase locking) to the TE (e.g., Frisina, 2001; Sayles et al., 2013) or TFS (e.g., Young and Sachs, 1979). In the healthy auditory system, both TE and TFS cues, and their comparison across different places on the basilar membrane, are used for speech identification (for a review, see Moore, 2014).

Aging in the absence of elevated audiometric thresholds does not seem to have a significant negative effect on frequency selectivity, as measured using psychophysical tuning curves or the notched-noise procedure (Lutman et al., 1991; Peters and Moore, 1992; Sommers and Humes, 1993; Gifford and Bacon, 2005). Although some studies reported a widening of the auditory filters with increasing age (Patterson et al., 1982; Glasberg et al., 1984), the older participants in those studies were either not audiometrically screened or had higher audiometric thresholds than the younger participants. Since elevated audiometric thresholds have been shown to be associated with greater auditory filter bandwidths (Moore, 2007), hearing loss most likely confounded the results. Given that the aim of the present study was to compare young and older participants with matched audiograms, measures of frequency selectivity were not included. Rather we focussed on measures of sensitivity to TE and TFS, based on behavioral (Pichora-Fuller and MacDonald, 2008; Moore, 2014) and neurophysiological (Walton et al., 1998; Clinard et al., 2010) data suggesting that aging negatively affects the processing of TE and TFS information.

Several studies of speech identification have used a signal-processing technique called vocoding (Dudley, 1939) to disrupt TFS information and reduce spectral cues, while substantially preserving information in the TE. These studies have shown that TE information in a few spectral bands can be sufficient for good identification of speech in quiet (Van Tasell et al., 1987; Shannon et al., 1995; Lorenzi et al., 2000). Modulation frequencies in the range 4–16 Hz seem to be especially important for the identification of speech in quiet (Drullman et al., 1994a,b). However, when speech is presented against interfering speech maskers, both slower and faster TE cues, associated respectively with prosodic (Füllgrabe et al., 2009) and fundamental frequency (Stone et al., 2008) information, become important for identification. Older listeners seem less able to use these complex TE patterns across different places on the basilar membrane to achieve speech identification (Souza and Boike, 2006; Schvartz et al., 2008; Sheldon et al., 2008), possibly due to reduced sensitivity to TE cues. Such a reduction should not be due to the presence of reduced hearing sensitivity in some of those listeners since, when the audibility of the stimuli is controlled for, hearing-impaired listeners have either similar (Moore and Glasberg, 2001) or better (Füllgrabe et al., 2003) TE sensitivity than normal-hearing listeners. Also, several studies using older listeners with nearly normal audiograms reported significant age-related decrements in the detection of sinusoidal amplitude modulation (SAM) imposed on pure-tone (He et al., 2008) or noise carriers (Takahashi and Bacon, 1992; Kumar and Sangamanatha, 2011). However, the results of the studies using noise carriers might have been affected by higher audiometric thresholds (especially in the high-frequency range) for the older than the younger participants, resulting in a smaller audible carrier bandwidth, which negatively affects SAM detection (Eddins, 1993). Here, TE sensitivity was assessed by measuring thresholds for detection of SAM presented over a range of modulation frequencies.

TFS information does not seem to be critical for the identification of speech in quiet. It may be more important when background sounds are present, perhaps by providing cues for auditory scene analysis (segregation of target and background sounds), such as sound-lateralization and voice-pitch cues (for an overview, see Moore, 2014). It has been argued that people with hearing loss have reduced TFS sensitivity (Smoski and Trahiotis, 1986; Hopkins and Moore, 2011), resulting in lower speech intelligibility (Lorenzi et al., 2006). An increasing number of studies (Pichora-Fuller and Schneider, 1992; Ross et al., 2007; Grose and Mamo, 2010; Moore et al., 2012a; Füllgrabe, 2013; Whitmer et al., 2014) indicate that age *per se* may also negatively affect TFS sensitivity. However, most studies used young and older participants whose audiograms were not matched, which could have led to the observed differences. Here, TFS sensitivity was assessed monaurally and binaurally for audiometrically matched YNH and ONH participants.

The decision to conduct a cognitive assessment, in addition to psychoacoustic tasks, was motivated by the general assumption that top-down cognitive processes are involved in speech processing (Eysenck and Keane, 2000) and empirical evidence that many cognitive functions decline with age (e.g., Baltes and Lindenberger, 1997; Park et al., 2002). Akeroyd (2008) reviewed 20 studies investigating the link between performance on SiN and cognitive tasks. He concluded that, while cognition was generally linked to SiN identification, there was no single cognitive test that consistently showed such an association. Across-study differences in sample characteristics (age, hearing status, general cognitive functioning), speech material (syllables, words, sentences), and listening conditions (interfering noise or babble), as well as their interactions, might account for the observed discrepancies. Here, we used a battery of cognitive tests to investigate the role of particular cognitive abilities (such as memory, attention, and processing speed) and general cognitive functioning in SiN processing.

Finally, the choice of two types of speech tasks (closed-set phoneme identification without semantic or syntactic context vs. open-set sentence identification with linguistic context) reflects an attempt to capture different levels of speech processing (Pickett, 1999). Varying the listening conditions (e.g., in quiet, in reverberation, in the presence of different types of maskers) was meant to modulate the perceptual and cognitive load (Mattys et al., 2009). Here, identification performance was assessed for maskers producing little informational masking (Durlach et al., 2003) in the absence of reverberation, and for maskers producing considerable informational masking in the presence of reverberation.

In summary, the present study aimed to measure possible deficits in the ability to identify speech in quiet and in background sounds that occur with increasing age, in spite of the absence of hearing loss as measured by the audiogram. The aims were to establish: (1) the existence and magnitude of such deficits; (2) the degree of awareness of the deficits; and (3) the extent to which the deficits were associated with declines in auditory and cognitive processing.

## Materials and methods

A discussion of methodological issues related to this study is provided in the Supplementary Material: *Methodological issues*.

### Participants

Potential participants were recruited from the Cambridge (UK) area through age-targeted (18–29 years or ≥ 60 years) advertisements posted in public spaces (e.g., doctors' surgeries) and appeals to social and community clubs. Nine younger (six females) and 21 older (20 females) native English speakers were retained for this study based on them having normal hearing sensitivity as defined by the audiometric criteria given below. The mean age of the YNH participants was 23 years (standard deviation, *SD* = 3; range = 18–27) and that of the ONH participants was 67 years (*SD* = 5; range = 60–79). All ONH participants completed the Mini Mental State Examination (Folstein et al., 1975) to screen for cognitive impairment, generally taken as indexed by scores < 24/30 points. All obtained full marks, bar one, who scored 29; this observation is consistent with population-based norms for 65–69-year olds with at least some university education (Crum et al., 1993). The number of years of formal education was, on average, 16.2 (*SD* = 2.0) and 16.8 (*SD* = 1.9) for the YNH and ONH groups, respectively. An independent-samples *t*-test showed that the age-group difference was not significant [*t*_(28)_ = 0.712, *p* = 0.482; two-tailed]. However, given that this proxy measure of cognitive ability is likely biased by cohort effects (ONH participants could have been prevented by historical circumstances and societal attitude toward education from attaining further education, while some YNH participants still had not completed their education), the two non-verbal sub-tests of the Wechsler Abbreviated Scale of Intelligence (WASI; Wechsler, 1999), Block Design and Matrix Reasoning, were also used to confirm the equivalence of the groups in terms of general cognitive functioning. Performance on the two tests can be combined into a performance IQ (see WASI manual). While the mean raw scores for the two tests differed across age groups (see Result section), the corresponding performance IQ scores (incorporating an age correction), were 123 (*SD* = 7) for the YNH and 122 (*SD* = 11) for the ONH group. This corresponds to the 92nd and 88th percentiles, respectively. According to an independent-samples *t*-test, the difference in age-corrected performance IQ was not significant [*t*_(28)_ = −0.441, *p* = 0.663; two-tailed]. Individual differences in mental functions show a high stability across the human lifespan (Deary et al., 2000; Gow et al., 2011) and the inter-individual variability in various cognitive abilities does not seem to increase with age (e.g., Salthouse, 2004, 2012). Under these circumstances, it is reasonable to assume that both age groups were sampled from the same cognitively high-functioning stratum of the underlying young and older populations.

All participants were fully informed about the aims of the study (approved by the local Cambridge University Ethics committee), provided written consent, and received monetary compensation for their participation.

#### Audiological assessment of hearing

**Figure 1:**
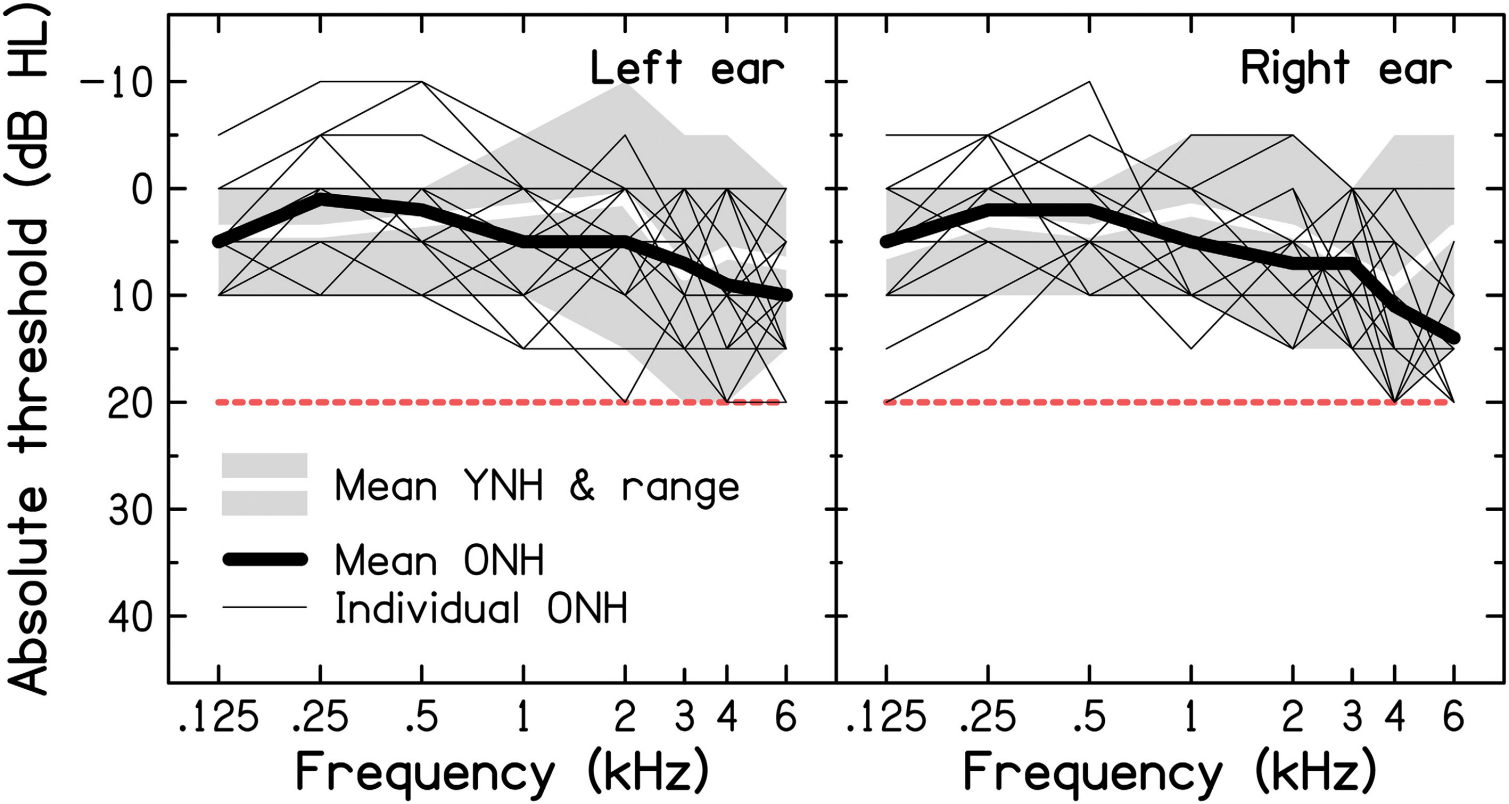
**Results of pure-tone air-conduction audiometry for the left (left panel) and right ears (right panel) of the nine YNH and 21 ONH participants**. The thin and thick black lines represent the individual and mean audiograms of the ONH participants. The thick white lines and associated light-gray shaded areas represent the mean audiograms and ranges of audiometric thresholds for the YNH participants, respectively. The dashed red line indicates the audiometric inclusion criteria used in the present study.

Following a clinical interview (including questions about difficult listening situations), pure-tone air-conduction audiometry was conducted using a Grason-Stadler GSI61 Clinical Audiometer with TDH-50P headphones, following the procedure recommended by the British Society of Audiology (BSA, 2004). In this study, normal hearing sensitivity was defined as audiometric thresholds ≤ 20 dB Hearing Level (HL) in both ears at octave frequencies between 0.125 and 4 kHz, as well as at 3 and 6 kHz. Audiograms for the YNH and ONH participants are shown in Figure 1 (for a comparison with audiometric thresholds found in a sample of older volunteers with self-reported normal hearing, see Supplementary Material: *Audiometric screening results for older volunteers with self-reported normal hearing*). Mean audiometric thresholds for the two age groups (thick lines) were very similar at all frequencies in both ears (the grand pure-tone average, PTA_0.125−6 kHz_, was 5.1 and 6.1 dB HL for the YNH and ONH groups, respectively), except for the right ear at 6 kHz, where the threshold for the ONH group was higher by 8.5 dB. The mean audiometric threshold did not differ significantly across groups, as shown by an independent-samples *t*-test [*t*_(28)_ = 0.808, *p* = 0.426; two-tailed].

#### Subjective assessment of hearing

Paper-and-pencil versions of two self-report inventories, routinely used for the assessment of hearing-aid benefit, were administered to all participants to assess their hearing abilities in various everyday listening conditions. At the time of questionnaire completion, none of the participants was aware of the outcome of the audiometric assessment.

**Figure 2:**
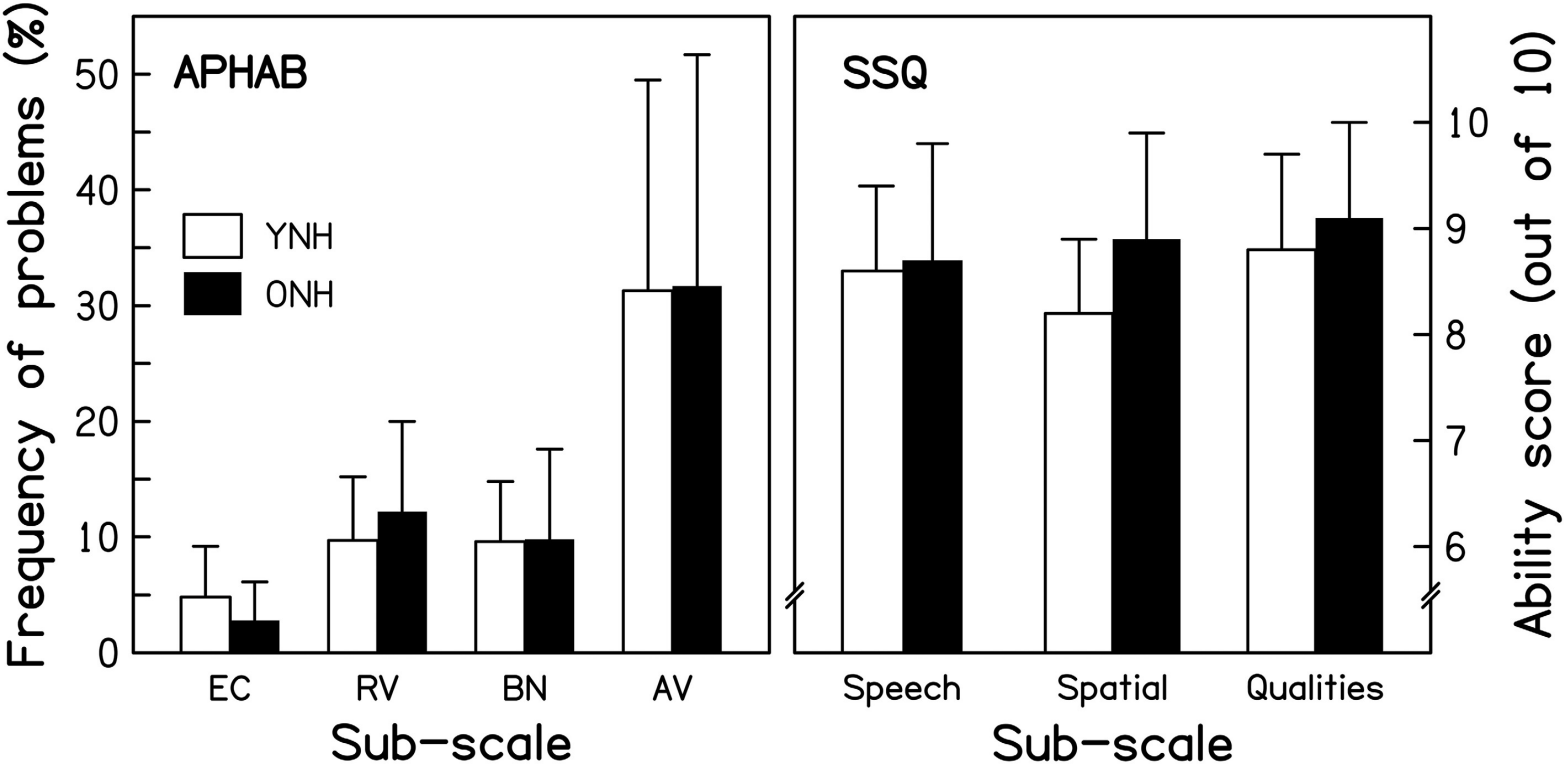
**Scores for the YNH (open bars) and ONH (filled bars) participants for two questionnaires**. For the Abbreviated Profile of Hearing Aid Benefit (APHAB; left panel), responses in terms of frequency of experiencing the described problems are averaged for each of four sub-categories: Ease of communication (EC), Reverberation (RV), Background noise (BN), and Aversiveness (AV). For the Speech, Spatial, and Qualities of hearing scale (SSQ; right panel), responses on an 11-point scale (0–10, with greater scores reflecting less disability) are averaged for the sub-categories of Speech hearing (14 questions), Spatial hearing (17 questions), and Qualities of hearing (19 questions). Note that more hearing difficulties are indicated by taller and smaller bars in the left and right panels, respectively.

***The Abbreviated Profile of Hearing Aid Benefit***. The Abbreviated Profile of Hearing Aid Benefit (APHAB; Cox and Alexander, 1995) is a questionnaire composed of 24 short statements (e.g., “I can understand conversations even when several people are talking.”). Respondents are asked to estimate how frequently they experience problems in the described situation, by selecting one of seven ordinal response alternatives, ranging from “Always” (=99%) to “Never” (=1%). Sub-scale scores are computed for Ease of Communication (EC), Reverberation (RV), Background Noise (BN), and Aversiveness (AV) by averaging across the responses to six statements for each sub-scale. The average APHAB scores for the two age groups are shown in the left panel of Figure 2. The frequency of experiencing problems was very similar for the two groups, as confirmed by a mixed-design repeated-measures analysis of variance (ANOVA) with Age group as the between-subjects factor and APHAB sub-scale as the within-subjects factor. The effect of Age group was not significant [*F*_(1, 28)_ = 0.008, *p* = 0.930], nor was the Age group^*^APHAB sub-scale interaction [*F*_(1.229, 34.417)_ = 0.222, *p* = 0.691]^1^.

***The Speech, Spatial, and Qualities of hearing scale***. The Speech, Spatial, and Qualities of hearing scale (SSQ; Gatehouse and Noble, 2004) is a 50-item questionnaire developed to assess how effectively auditory information is being processed in a variety of everyday listening situations. Unlike the APHAB, it includes situations explicitly involving auditory scene analysis and cognitive abilities, such as focusing on one sound source in the presence of others, or attending to multiple sound sources simultaneously. One item was excluded from the original questionnaire since it was only applicable to hearing-aid users. For each of the remaining items, respondents are asked to estimate their (dis)ability in performing an auditory-based “activity” by selecting a number on an 11-point response scale, ranging from “0” (= complete disability) to “10” (= no disability). Each item is associated with one of three sub-scales: Speech hearing (14 items; e.g., “Can you have a conversation in the presence of someone whose voice is the same pitch as that of the person you're talking to?”), Spatial hearing (17 items; e.g., “Do you have the impression of sounds being exactly where you would expect them to be?”), and other Qualities of hearing (18 items; e.g., “Do you find it easy to recognize different people you know by the sound of each one's voice?”). Average ability scores for the two groups are shown in the right panel of Figure 2 for each of the sub-scales. Ratings were very similar for the two groups, as confirmed by a repeated-measures ANOVA that showed a non-significant effect of Age group [*F*_(1, 28)_ = 1.097, *p* = 0.304] and a non-significant Age group^*^SSQ sub-scale interaction [*F*_(2, 56)_ = 1.506, *p* = 0.231].

### Equipment

For all auditory tasks (unless otherwise stated; see Section Assessment of sensitivity to TE information), stimuli were played with 16-bit precision through a Lynx L22 soundcard hosted in a PC, under control of custom-written software in Matlab or VisualBasic. The sampling frequency was dependent on the task but was at least 16 kHz. The soundcard output signal was buffered by a Mackie 1202-VLZ PRO mixing desk, and delivered over Sennheiser HDA580 headphones to the participants, who were seated in a sound-attenuating booth. Depending on the task, response entry was made via either a mouse click on virtual buttons displayed on a computer screen, a manual button press, or orally.

For the cognitive tests, the experimenter sat with the participant in a large sound-attenuating booth or a quiet room. Depending on the test administered, participants gave their responses either orally or manually.

### Speech tasks

#### Consonant identification

Bisyllabic vowel-consonant-vowel (VCV) stimuli with 21 different consonants were used. An /a/ was used for the initial and final vowels. The consonants were /p, t, k, b, d, g, f, θ, s, ∫, h, v, z, r, l, j, w, t, dj, n, m/. Four utterances of each VCV were spoken by a female talker with a standard British accent, with the emphasis on the second syllable. Recordings were made in an anechoic room with 16-bit quantization and a 44.1-kHz sampling rate, later digitally down-sampled to 16 kHz.

Consonant identification was assessed using a 1-interval, 21-alternative forced-choice procedure. In each run, all 21 VCVs were presented once in random order. Following the presentation of a VCV, the participant indicated which consonant had been heard by selecting one of 21 virtual buttons, each labeled with the orthographical representation of one of the consonants in a meaningful CV word.

VCVs were presented in quiet and in three types of background masker whose long-term average spectrum was shaped to be the same as that of the VCVs: (1) unmodulated^2^ noise; (2) noise with 100%, 5-Hz SAM^3^ applied to its TE; and (3) noise with 100%, 80-Hz SAM applied to its TE. Masked speech testing, for example during speech audiometry (see Katz et al., 2009), is traditionally performed using unmodulated speech-shaped noise. Here, performance was also assessed with modulated noises, because it has been suggested that age-related speech-identification deficits might be exacerbated when the background has such fluctuations (Takahashi and Bacon, 1992; Stuart and Phillips, 1996; Dubno et al., 2002). The choice of the 5-Hz SAM frequency was motivated by the finding of Füllgrabe et al. (2006) that, compared to consonant identification in the presence of an unmodulated noise, the largest improvement in performance was observed for a noise with an SAM frequency between 4 and 16 Hz. This release from masking is believed to reflect the ability to take advantage of the minima in the fluctuating noise to detect speech cues, a phenomenon referred to as “dip listening” (e.g., Cooke, 2006; Füllgrabe et al., 2006). A higher modulation frequency was also used to test the hypothesis that older listeners show less masking release when the masker has only short temporal dips, due to decreased temporal resolution (Takahashi and Bacon, 1992; He et al., 2008; Kumar and Sangamanatha, 2011) or increased susceptibility to forward masking (Dubno et al., 2002; Gifford et al., 2007). Noises were ramped on and off using a 50-ms raised-cosine function, and started and ended synchronously with the VCVs. VCVs were presented at 65 dB Sound Pressure Level (SPL), approximating normal conversational speech levels (Olsen, 1998). The noise level was varied in 4-dB steps to give signal-to-noise ratios (SNRs) of −2 to −14 dB for the unmodulated noise, and −6 to −18 dB for the SAM noises. All stimuli were lowpass filtered at 6 kHz (with frequency components above 6.125 kHz attenuated by at least 100 dB) to produce zero audibility above that frequency for both age groups. Stimuli were presented diotically.

Practice was given prior to data collection using four training runs, drawn from each of the four conditions (quiet, unmodulated noise at −2-dB SNR, 5-Hz SAM noise at −6-dB SNR, and 80-Hz SAM noise at −6-dB SNR). Visual feedback and the possibility of repeating a given VCV were provided, and participants were encouraged to use the repeat option whenever necessary. No feedback was provided during the test phase, in which the 13 experimental conditions were presented twice, once in each of two test blocks separated by a break. Each test block started with the speech-in-quiet condition. In the first block, identification was then assessed using the unmodulated, the 5-Hz SAM, and the 80-Hz SAM noise conditions; for each noise type, the SNR conditions were presented in descending order. In the second block, the noise conditions were presented in reverse order to balance possible learning and fatigue effects.

#### Sentence identification

Target sentences were taken from the main corpus of the Adaptive Sentence Lists (MacLeod and Summerfield, 1990) and comprised 18 lists, plus four “trash” lists (the sentences in these were not as well matched for difficulty as for the other lists). Each list contained 15 sentences. Sentences (mean duration = 1510 ms) had three key words and a simple syntactic structure, and were somewhat predictable (e.g., “*They moved* the *furniture*”). Sentences were spoken by a male talker with a standard British accent, and presented either in quiet or against two interfering male talkers (one with a British and one with a soft Australian accent), reading from prose passages in a normal conversational manner (Moore et al., 2008). Pauses exceeding 300 ms were truncated “by hand,” and the two interfering talkers were added together at the same root-mean-square (rms) level. To simulate real-world listening conditions (containing reverberation and spatially separate sound sources), the target and interfering speech were played through one of two Tannoy Precision 8D self-powered loudspeakers to a KEMAR head-and-torso manikin in a moderately reverberant lecture theater (RT60 = 0.67, 0.67, 0.54, 0.56, 0.53, 0.53, and 0.53 s for 1/3-octave-wide bands centered at 0.125, 0.25, 0.5, 1, 2, 4, and 8 kHz, respectively; Moore et al., 2010). The loudspeakers were positioned at ±60° relative to KEMAR's sagittal plane, and at a distance of 1.5 m. Recordings with 16-bit quantization and a 44.1-kHz sampling rate were obtained separately from the two ears, and then processed off-line (including an inverse diffuse field correction for Kemar's meatal response). For the masked conditions, left- and right-ear recordings of the target speech played through one loudspeaker were combined with left- and right-ear recordings of the interfering speech, respectively, when played through the same loudspeaker (giving rise to a co-located percept of the talkers) or through the other loudspeaker (giving rise to spatially separate percepts of the talkers). Target sentences were inserted into randomly selected 3-s excerpts of the interfering-talker mixture. The onset of the target sentences varied randomly from 0 to 500 ms relative to the onset of the interfering speech. The level of the target speech was fixed at 65 dB SPL and the level of the interfering speech was varied in 4-dB steps to give SNRs of −2 to −18 dB. All stimuli were lowpass filtered at 6 kHz (with attenuation of at least 100 dB above 6.125 kHz).

The task was to repeat orally as many words as possible from each target sentence. Response time was unlimited. The trash lists were used to present six practice conditions: quiet, co-located at −2 and −6 dB SNR, and spatially separate at −6, −10, and −14 dB SNR. Lists 1–18 from the main corpus were used for the 16 experimental conditions obtained by combining the three factors (Masker location, SNR, and Target position), plus two quiet conditions, one for each target position. The order of presentation of conditions was counterbalanced using a Latin-square design.

### Supra-threshold psychoacoustic tasks

#### Assessment of sensitivity to TE information

The threshold for detecting SAM imposed on a 4-kHz sinusoidal carrier was measured using a 3-interval, 3-alternative forced-choice procedure with feedback. On each trial, three consecutive 1-s observation intervals were presented, separated by 415-ms silences. One interval, selected at random, contained the SAM tone (the “target”) and the other two intervals (the “standards”) contained the unmodulated carrier. All stimuli had the same rms level. The task was to indicate the interval containing the target.

Modulation frequencies (*f_m_*) of 5, 30, 90, and 180 Hz were used to characterize the temporal-modulation-transfer function (TMTF; Viemeister, 1979), covering the three types of TE-based percepts, namely loudness fluctuations, roughness, and residue pitch (see Figure 2 in Joris et al., 2004). The modulation depth (*m*) at the start of a run was set to 0.5, 0.6, 0.6, and 0.7 for the four values of *f_m_*, respectively. The value of *m* was changed adaptively using a 3-down, 1-up stepping rule, estimating the 79%-correct point on the psychometric function (Levitt, 1971). The initial step size was a factor of 1.78, and the step size was reduced to a factor of 1.26 after the first two reversals. After a total of 70 trials, the run was terminated, and the geometric mean of the values of *m* at the last eight reversals was taken as the threshold estimate.

A 4-kHz carrier was used to ensure that TE and not spectral cues were used to perform the task; spectral sidebands produced by the SAM would not have been resolved even for *f_m_* = 180 Hz (Kohlrausch et al., 2000). The level of the carrier was set to 30 dB Sensation Level (SL), based on the participant's absolute thresholds for a 4-kHz pure tone, measured at the beginning of the test. This limited the spread of the excitation pattern, thus minimizing “off-frequency listening,” which has been shown to affect TE processing (Füllgrabe et al., 2005).

Prior to data collection, participants received practice in the form of one threshold run for each value of *f_m_*. The test phase consisted of two repeated measures for each *f_m_*, administered first in one order (30, 180, 5, and 90 Hz), and, after a break, in the reverse order.

All stimuli were digitally generated using a PC-controlled Tucker-Davis-Technologies (TDT) system with a 16-bit digital-to-analog converter (DD1, 50-kHz sampling rate), lowpass filtered at 20 kHz (Kemo VBF8, mark 4), attenuated (TDT P4), passed through a headphone buffer (TDT HB6), and delivered diotically at 65 dB SPL.

#### Assessment of sensitivity to TFS information

The ability to detect changes in the temporal fine structure (TFS) of tones, within the same ear and across ears, was assessed using two tests developed by Moore and colleagues (Moore and Sek, 2009; Hopkins and Moore, 2010). In both tests, a 2-interval, 2-alternative forced-choice procedure with feedback was used. On each trial, two consecutive intervals were presented, separated by 500 ms. Each interval contained four consecutive 400-ms tones, separated by 100 ms. All tones were shaped using a 20-ms raised-cosine function. In one interval, selected at random, the TFS of all tones was identical (the standard). In the other interval (the target), the first and third tones were the same tones as in the standard interval while the second and forth tones differed in their TFS. Listeners with “normal” TFS sensitivity perceive the change in TFS as a variation either in pitch (in the monaural TFS test; see below) or in lateralization (in the binaural TFS test; see below), and thus can identify the interval containing the changing tone sequence when large TFS differences are used. Initially, the difference in TFS between tones was set to the maximum value possible, without producing ambiguous percepts. The manipulated variable was adaptively adjusted, using a 2-down, 1-up stepping rule to estimate the 71%-correct point on the psychometric function (Levitt, 1971). The value of the manipulated variable was changed by a factor of 1.25^3^ until the first reversal, then by a factor of 1.25^2^ until the next reversal, and by a factor of 1.25 thereafter. After eight reversals, the run was terminated and the geometric mean of the values at the last six reversals was taken as the threshold estimate. When the SD of the log values at the last six reversals exceeded 0.2, indicating high variability, the estimate was discarded and a new run was conducted. If the adaptive procedure called for values exceeding the maximum more than twice during a run, the adaptive procedure was terminated, and 40 constant-stimuli trials were presented with the value fixed at its maximum. Two valid threshold estimates were obtained for each condition. Practice in the form of at least one threshold run for each of the four monaural and two binaural conditions was provided prior to data collection.

***Monaural TFS test***. Monaural TFS sensitivity was assessed using the TFS1 test (Moore and Sek, 2009). Participants were asked to discriminate harmonic complex tones, with a fundamental frequency *F*0, from similar tones in which all components were shifted up by the same amount in Hz, resulting in inharmonic complex tones. The frequency shift was the manipulated variable, and it was initially set to 0.5*F*0. The tones had the same envelope repetition rate, but different TFS. The starting phases of the components in each tone were random, resulting in random differences in the shape of the TE of the complex tones and preventing TE shape from being used as a cue. All tones were passed through a bandpass filter (with a bandwidth of 1*F*0 and slopes of 30 dB/octave), centered on 11*F*0. Since the auditory system does not resolve harmonics above the 8th (Plomp, 1964; Moore and Ohgushi, 1993), all components in the passband were unresolved and, consequently, differences in excitation pattern for the two tones were minimal (Hopkins and Moore, 2007). Two *F*0s, 91 and 182 Hz, were used, corresponding to filter center frequencies of 1 and 2 kHz, respectively. To mask combination tones and components falling on the skirts of the bandpass filter, threshold equalizing noise (TEN; Moore et al., 2000) was presented. Its level/ERB_N_ (Moore, 2012) was 15 dB below the overall level of the tones. The TEN was gated on and off with 20-ms raised-cosine ramps and started 300 ms before the first tone in the first interval and ended 300 ms after the last tone in the second interval. The overall level of the complex tones was set to 30 dB SL, based on absolute-threshold measurements for pure tones at the two filter center frequencies. Each ear was tested separately.

***Binaural TFS test***. Binaural TFS sensitivity was assessed using the TFS-LF test (Hopkins and Moore, 2010). Participants were asked to discriminate binaurally presented pure tones with identical phases at the two ears (perceived as emanating from a central position inside the head) from tones with a phase shift between the ears (perceived as being lateralized toward one ear). The interaural phase shift (Δϕ) was the manipulated variable. Two frequencies, 0.5 and 0.75 kHz, were used. For both, Δϕ was initially set to 180°. All tones were gated on and off synchronously in the two ears to avoid the use of interaural differences in TE to perform the task. The level of the tones was set to 50 dB SPL in each ear.

### Cognitive tasks

With the advent of cognitive hearing science (e.g., Arlinger et al., 2009; Rönnberg et al., 2011) new interest has been sparked concerning the role of cognition in normal and pathological speech perception. An increasing number of studies have included some form of cognitive assessment. However, the number and diversity of cognitive tests have generally been small, and their choices have not always been explicitly motivated. In keeping with past attempts to assess more systematically the relationship between cognitive functioning and speech perception (e.g., Van Rooij et al., 1989; Van Rooij and Plomp, 1990; Jerger et al., 1991; Humes et al., 1994), a large number of cognitive abilities was investigated in the present study. The reasons for selecting the specific cognitive tasks in terms of their relationship with age and speech processing are discussed in the Supplementary Material: *Relationship between cognitive-task performance, age, and speech intelligibility*.

#### Digit span test

The Digit Span (DS) test, taken from the Wechsler Adult Intelligence Scale—Third Edition (WAIS-IIIUK; Wechsler, 1997), is assumed to assess short-term-memory (STM) capacity (i.e., the temporary storage of information) and working-memory (WM) capacity (i.e., storage plus processing of information), using the Digits Forward (DS-F) and Digits Backward (DS-B) tests, respectively. In the former, digit sequences of increasing length (from 2 to 9 digits) are presented verbally at one digit per second for immediate verbal recall. Two trials for each sequence length are presented. The task is discontinued after two incorrect answers for a given sequence length. The final DS-F score used here corresponded to the sum of recalled digits for all entirely correctly reported sequences; the maximum total score was 88. In the DS-B test, digit sequences of increasing length (containing 2 to 8 digits) are presented, but the digits have to be recalled in reverse order (i.e., from last to first). The final DS-B score was computed in the same way as the DS-F score; the maximum total score was 70. An initial practice trial was given for each test.

#### Reading span test

The Reading Span (RS) test, originally developed by Daneman and Carpenter (1980), is one implementation of a complex span test (Conway et al., 2005), designed to assess the key properties of the limited-capacity working-memory (WM) system, namely memory storage and information processing (Baddeley, 1992). Here, a computerized version (Rönnberg et al., 1989) of the RS test of Baddeley et al. (1985) was used, in which short, grammatically correct sentences were displayed in a word-by-word fashion on a computer screen (e.g., “The ball—bounced—away”) at a rate of one word every 800 ms. A 1750-ms silent interval separated the end of one sentence from the beginning of the next sentence. Half of the sentences were sensible while the others were absurd (e.g., “The pear—drove—the bus”). Sentences were arranged in three sets of three, four, five, and six sentences, and presented in order of increasing length. All sets were administered, irrespective of the participant's performance. The task was to read aloud each sentence and then to indicate by a verbal “yes/no” response if the sentence made sense or not (processing component of WM). At the end of each set, the participant was instructed to recall in correct serial order either the first or the last word of each sentence (storage component of WM). The requested recall position (first or last) varied pseudo-randomly (with first-word recalls in half of the sets) but was identical for all participants. Prior to testing, practice was given in the form of one three-sentence set, which was repeated if necessary until the instructions were clearly understood. To assess whether participants traded performance on the semantic-judgment task in favor of the recall task in an age-dependent manner, the number of errors on the semantic-judgment task was analyzed: there was no significant difference between the two age groups [*t*_(27)_ = −0.528, *p* = 0.602; two-tailed]. Following others (Lunner, 2003; Sörqvist and Rönnberg, 2012), the percentage of first and last words correctly recalled in any order out of the total number of words to be recalled (i.e., 54) was taken as an indicator of WM capacity.

#### Test of everyday attention

The Test of Everyday Attention (TEA; Robertson et al., 1994) is a neuropsychological test designed to assess the integrity of different, functionally independent attentional systems (Posner and Petersen, 1990). Using principal-component analysis and cross-validating with established tests of attention, Robertson et al. (1996) identified four putative cognitive processes probed by the following eight sub-tests of the TEA:

The *Map Search* and *Telephone Search* tests require the participant to visually search, as quickly as possible, for predetermined symbols, either on a map or in a telephone directory. The number of identified symbols and the time per symbol are recorded in the first and second tests, respectively. Performance on both tests indexes selective attention. In the *Elevator Counting* and *Lottery* tests, participants count the number of tones in sequences of varying length, and monitor a 10-min recording of lottery ticket numbers for winning numbers, respectively. Performance on these tests, and the *Telephone Search while Counting* test (in which participants perform the Telephone Search test while simultaneously counting the number of tones in a sequence), assesses sustained attention. In the *Visual Elevator* test, the participant counts the number of visual symbols in an ascending and descending order, following visual instructions. Time to completion for correct trials assesses attentional switching. The auditory analog of this test is the *Elevator Counting with Reversals* test, using tones of different pitches as items to be counted and also as instructions to count up or down. Performance on this test and on the *Elevator Counting with Distraction* test (which requires counting the number of tones in a sequence while ignoring interleaved distractor tones of a different frequency) indexes audio-verbal WM. Practice was provided for all sub-tests prior to testing according to the TEA instructions.

#### Trail making test

The two parts of the paper-and-pencil version of the neuropsychological Trail Making (TM) test (Reitan, 1955) were administered, following the protocol described by Bowie and Harvey (2006). In Part A, 25 encircled Arabic numerals (1–25), randomly distributed on a white sheet of paper, had to be connected in ascending order. The participants were instructed to complete the task as quickly and as accurately as possible, and time to completion was recorded. It is generally assumed that this part assesses psycho-motor speed and visual search (e.g., Crowe, 1998). In Part B, 12 Arabic numerals (1–12) and 12 letters (A-L) had to be connected in ascending order, alternating between numerals and letters (A-1-B-2-C-3, *etc*.). Keeping two mental sets in memory and switching between them requires additional executive control (Arbuthnott and Frank, 2000). Prior to test administration, participants completed shorter practice versions of each part.

Derived measures, for example the difference between completion times for the two parts (Part B—Part A; e.g., Sanchez-Cubillo et al., 2009) or the ratio of the two completion times (Part B/Part A; e.g., Lamberty et al., 1994) have been used to provide a “purer” estimate of executive control abilities. However, as pointed out by Verhaeghen and De Meersman (1998), age-group differences in difference scores are still confounded by age-related deficits in processing speed. Hence, we computed the normalized derived measure [(Part B—Part A)/Part A] to assess executive control.

#### Block design test

Block Design (BD) constitutes a standard measure of performance IQ in many test batteries of intelligence. It is assumed to measure spatial perception, visual abstract processing, and problem solving. Here, we used the BD version from the WASI (Wechsler, 1999), in which participants had to manually rearrange four or nine two-color blocks to replicate 13 target “designs” displayed on a series of test cards and presented in order of increasing difficulty. The two easiest designs were used as practice. Time to completion for each design was measured and transformed to a point score (from 0 to 7); designs completed after predefined cutoff times were scored as zero. The maximum total score was 71.

#### Matrix reasoning test

Matrix Reasoning (MR) is another standard test for measuring non-verbal abstract reasoning. Here, we used the version taken from the WASI (Wechsler, 1999), comprising 35 items, organized in order of increasing difficulty. Each item was composed of a matrix of geometric patterns with one element missing. The task was to choose from five response alternatives the one that best completes the matrix. The two easiest designs were used as practice. No time limit was imposed. The maximum total score was 35.

## Results and discussion

Age-group differences in sensitivity and performance were assessed, using independent-samples *t*-tests, and, in cases of the simultaneous manipulation of within-subjects factors, mixed-design repeated-measures analyses of variance (ANOVAs) with Age group as the between-subjects factor. To assess the strength of association between the various measures of supra-threshold auditory processing, cognitive abilities, and speech identification, Pearson product-moment correlation coefficients were computed for the entire group of participants (see table of all correlations in the Supplementary Material: *Grand correlation matrix for the combined group of young and older normal-hearing participants*), for the ONH participants alone, and for all participants with the effect of age partialled out. Finally, multiple regression analyses were conducted to quantify the relative contribution of different processing abilities to consonant and sentence identification.

### Speech tasks

Several authors (e.g., Dubno and Ahlstrom, 1997; Demeester, 2011) have highlighted the possibility that changes in the audiogram of a few dB may be associated with changes in speech perception in noise. This motivated the matching of audiograms for the two age groups used in the present study. The results presented in this section will mainly be compared to those for studies using fairly stringent definitions of normal audiograms (e.g., thresholds ≤ 25 dB HL over a wide range of frequencies) and using lowpass-filtered target speech to restrict the spectrum of the stimuli to the frequency range where audiometric thresholds were normal, or to studies where age groups were audiometrically matched.

Individual identification scores were transformed into rationalized arcsine units (RAUs, Studebaker, 1985) for statistical analyses. To ease interpretation, the averaged transformed data were transformed back to percentages for the presentation of the results in the figures and the text.

#### Consonant identification

**Figure 3:**
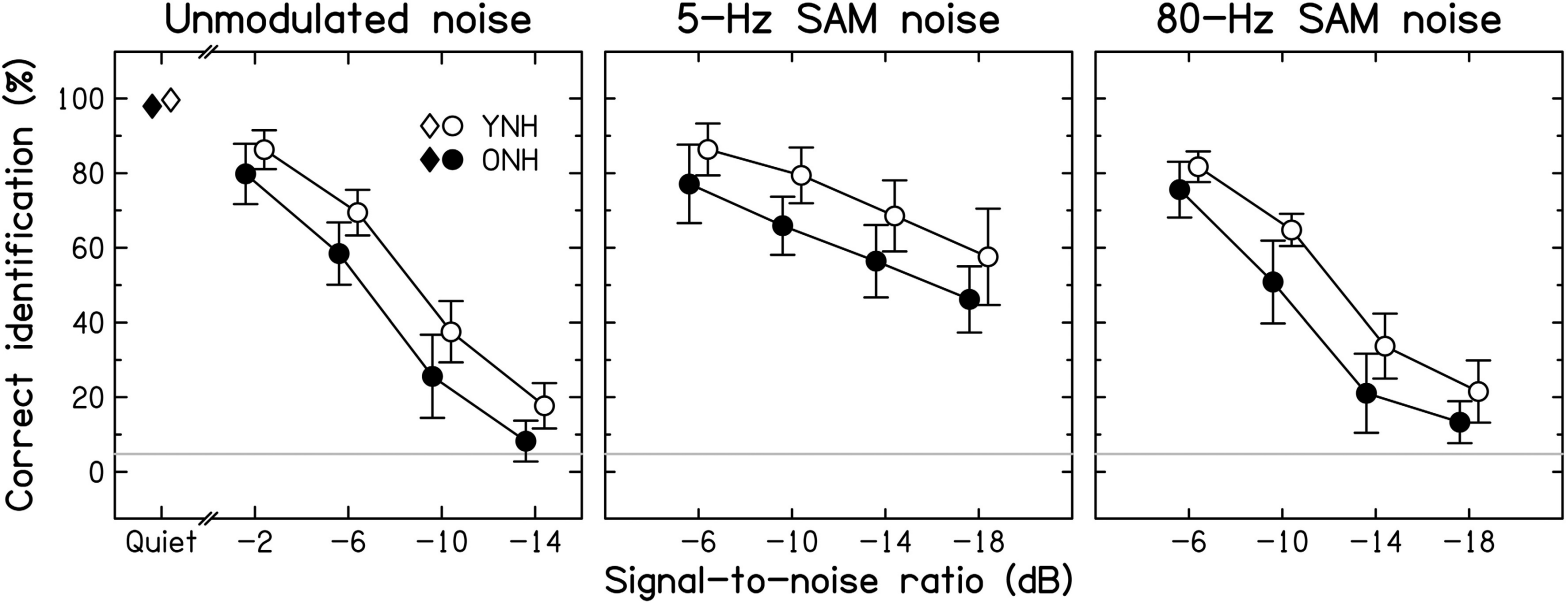
**Average consonant-identification performance in different listening conditions for YNH (open symbols) and ONH (filled symbols) participants**. Identification scores are given for the quiet condition (diamonds) and as a function of the signal-to-noise ratio (SNR) for the unmodulated, 5-Hz SAM, and 80-Hz SAM noise conditions (left, middle, and right panels, respectively). Here, and in the following figures, data points for the two groups are slightly displaced horizontally to aid visibility. Chance-level performance is indicated by the gray horizontal lines. Error bars represent ±1 SD.

***Intelligibility***. Group-mean consonant-identification scores are plotted in Figure 3 for the YNH (open symbols) and ONH (filled symbols) participants for speech in quiet (left-most symbols) and in the three noise types (different panels) as a function of SNR.

Consonant identification in quiet was near-perfect for both age groups, but, consistent with previous results (Gelfand et al., 1985; Gordon-Salant, 1986), the ONH participants made slightly but significantly more confusions than the YNH participants [group difference of 1.7% points; *t*_(28)_ = −2.051, *p* = 0.05; two-tailed].

Consistent with Gelfand et al. (1986), the addition of background noise resulted in a larger decrease in identification scores for the ONH than for the YNH participants. However, the effect of decreasing SNR was similar for the two groups. Contrary to the assumption that the effect of age is greater for temporally fluctuating backgrounds than for unmodulated backgrounds (Takahashi and Bacon, 1992; Stuart and Phillips, 1996; Dubno et al., 2002), the three background noises yielded age-associated decrements of similar sizes: age differences averaged across SNRs were 9.7, 11.6, and 10.2% points for the unmodulated, 5-Hz SAM, and 80-Hz SAM noise, respectively. Given the use of different SNR ranges, a separate ANOVA was conducted for each noise type. In all three cases, there were significant effects of Age group [*F*_(1, 28)_ = 16.027, 21.865, and 23.413, respectively, all *p* < 0.001] and SNR [*F*_(3, 28)_ = 603.483, 68.312, and 391.025, respectively, all *p* < 0.001], but the Age group^*^SNR interaction was not significant [*F*_(3, 28)_ = 1.002, 0.270, and 1.533, respectively, *p* = 0.396, 0.847, and 0.212, respectively]. Scores for the different noise types were correlated moderately to strongly across all participants (all *r* ≥ 0.612, all *p* < 0.001), indicating that participants tended to perform consistently poorly or well.

**Figure 4:**
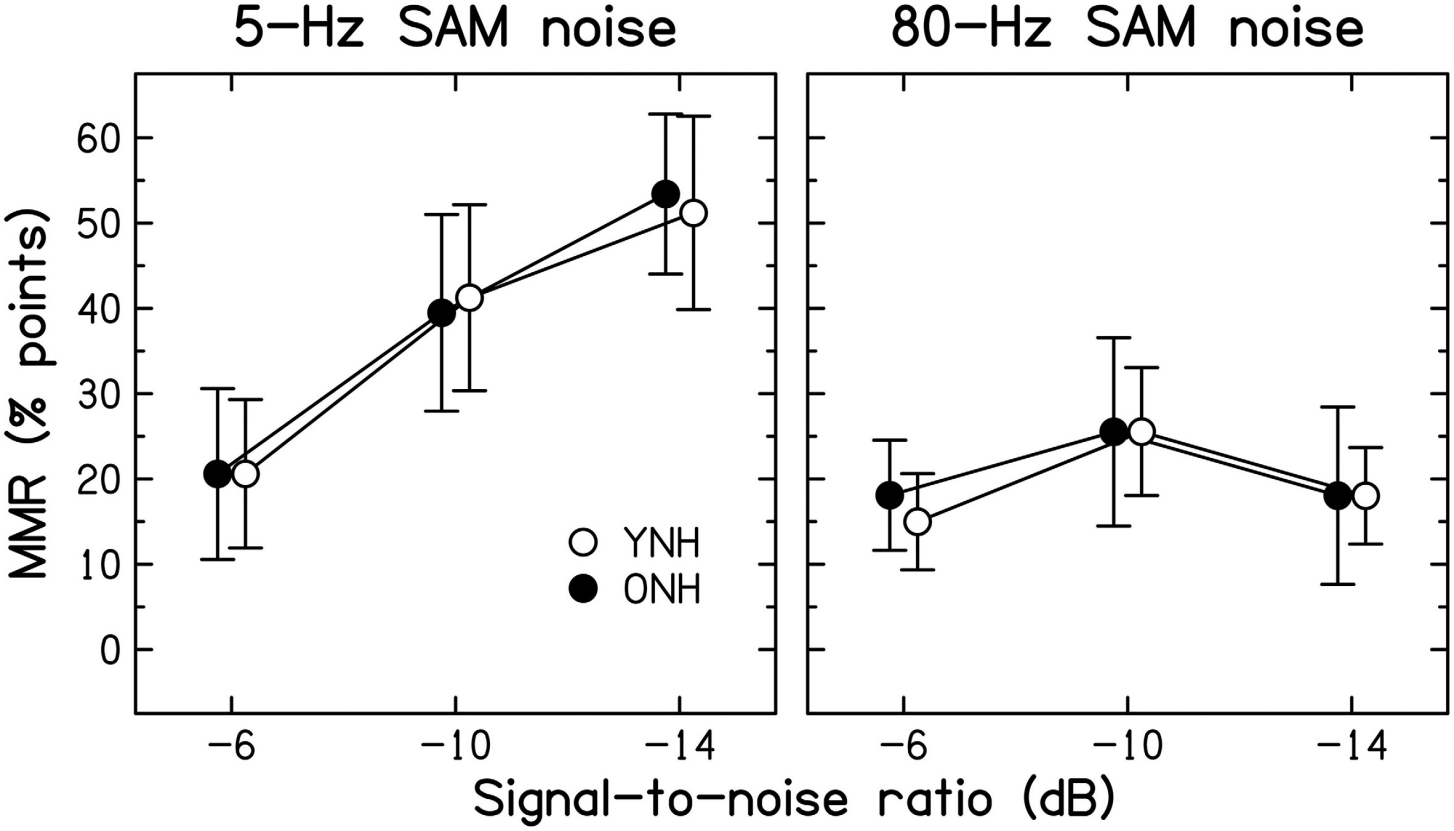
**Average amount of modulation masking release (MMR, in percentage points) for YNH (open symbols) and ONH (filled symbols) participants**. MMR is the difference in scores obtained using an SAM noise [SAM frequency = 5 Hz (left panel) or 80 Hz (right panel)] and an unmodulated noise.

***Modulation masking release***. The benefit derived from modulation of the background noise was computed as the difference in identification scores obtained at a given SNR with either of the two SAM noises and the unmodulated noise, and will be referred to as modulation masking release (MMR). The mean MMR values for 5-Hz and 80-Hz SAM are shown in Figure 4 for the two age groups.

Consistent with previous results for YNH participants (Füllgrabe et al., 2006), all participants showed more MMR for the lower than the higher SAM frequency. For the 5-Hz SAM noise, the amount of MMR increased monotonically with decreasing SNR, while it remained roughly constant for the 80-Hz SAM noise. Across all participants, identification performance for the unmodulated noise was negatively correlated with the amount of MMR (*r* = −0.464, all *p* = 0.010); participants who had low scores for unmodulated noise tended to show high MMR. However, this could be due to the fact that performance for the unmodulated noise enters into both quantities that were correlated.

Age-group differences in MMR were very small, barely exceeding 3% points. The main effects of SAM frequency [*F*_(1, 28)_ = 61.810, *p* < 0.001] and SNR [*F*_(1.629, 45.613)_ = 28.347, *p* < 0.001] were significant, as was the interaction between these two factors [*F*_(1.836, 51.398)_ = 18.363, *p* < 0.001]. However, neither the main effect of Age group [*F*_(1, 28)_ = 0.001, *p* = 0.972] nor any of the two- or three-way interactions involving this factor were significant (all *F* < 1, all *p* ≥ 0.607). These results indicate that the ability to “listen in the dips” does not decrease with increasing age, at least when peripheral hearing sensitivity is normal and matched across age groups. Some earlier investigations (Dubno et al., 2002, 2003; Grose et al., 2009) reported age-group differences in MMR. However, the older participants in those studies had higher audiometric thresholds than the younger participants, especially in the high-frequency range, and the bandwidth of the speech signals was not limited to the audiometrically normal range, which might explain the discrepancy between the present and previous results.

#### Sentence identification

**Figure 5:**
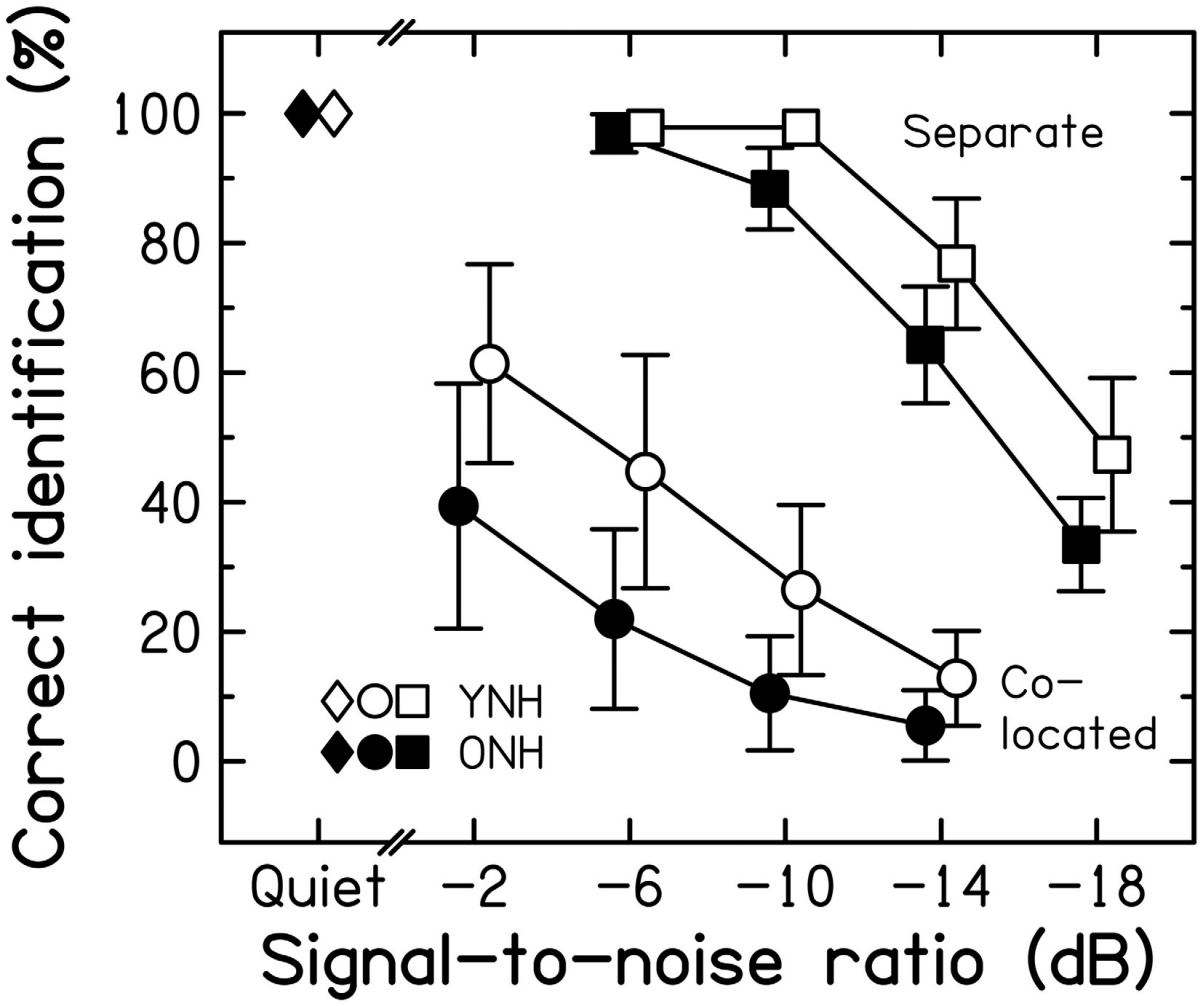
**Average speech-identification scores for three listening conditions for the YNH (open symbols) and ONH (filled symbols) participants**. Scores are given for the quiet condition (diamonds) and as a function of the SNR for the “co-located” (circles) and the “separate” conditions (squares). Error bars represent ±1 SD.

***Intelligibility***. Figure 5 presents group-mean scores for speech in quiet and in two-talker babble presented from the same spatial location as the target speech (“co-located”) or from a different spatial location (“separate”). The position of the target speech (localized toward the left or the right) was counterbalanced across conditions. However, a paired-samples *t*-test for the quiet conditions [*t*_(29)_ = −1.000, *p* = 0.326; two-tailed] and separate ANOVAs for the co-located [*F*_(1, 28)_ < 1, *p* = 0.957] and separate [*F*_(1, 28)_ = 3.609, *p* = 0.068] conditions revealed no significant effect of this factor. Hence, scores were pooled across the two target positions.

Unmasked speech identification was at ceiling and almost the same for the two age groups [*t*_(28)_ = −0.648, *p* = 0.522; two-tailed]. For the co-located condition, the ONH participants performed more poorly than the YNH participants over the entire range of SNRs. The age-group difference was 22% points at the highest SNR, and dropped to 7% points at the lowest SNR, most likely due to a floor effect. The main effects of SNR [*F*_(3, 84)_ = 97.151, *p* < 0.001] and Age group [*F*_(1, 28)_ = 17.154, *p* < 0.001] were significant but the SNR^*^Age group interaction was not [*F*_(3, 84)_ < 1, *p* = 0.433]. Performance was better for the separate than for the co-located condition for both groups. Scores were lower for the ONH than for the YNH participants for the three lowest SNRs; at the most favorable SNR, a ceiling effect was most likely responsible for the very similar scores for the two groups. There were significant main effects of SNR [*F*_(3, 84)_ = 592.247, *p* < 0.001] and Age group [*F*_(1, 28)_ = 19.200, *p* < 0.001] and a significant interaction [*F*_(3, 84)_ = 7.594, *p* < 0.001]. Performance in the two masking conditions was correlated strongly across all participants (*r* = 0.753, *p* < 0.001).

***Spatial masking release***. The improvement in speech identification produced by a difference in the spatial locations of target and masker signals (compared to the co-located case) is referred to as spatial masking release (SMR; e.g., Freyman et al., 1999). We quantified SMR by calculating the difference in scores for the separate and co-located conditions for the SNR that did not yield a floor effect for the former and a ceiling effect for the latter. For an SNR of −10-dB, the SMR values for the YNH and ONH participants were 84.6 and 86% points, respectively. This difference across age groups was not significant [*t*_(28)_ = 0.369, *p* = 0.715; two-tailed]. Thus, consistent with earlier investigations (Gelfand et al., 1988; Li et al., 2004; Singh et al., 2008; Cameron et al., 2011), these results provide no evidence to support the idea that the ability to use spatial separation between target and interfering speech declines with age when the audiogram is normal. This is surprising given that ONH participants have been shown to be less sensitive than YNH participants to inter-aural time differences (ITDs; Ross et al., 2007; Grose and Mamo, 2010; Füllgrabe, 2013; see also Section *Assessment of sensitivity to TFS information*). However, the potency of ITD cues in the physiological range in inducing sequential stream segregation does not seem to be affected in those listeners (Füllgrabe and Moore, 2014), and the listening conditions used here afforded additional cues (e.g., monaural spectral cues and interaural intensity differences) that contributed to SMR (Singh et al., 2008). The processing of these cues seems to be relatively unaffected by aging (Herman et al., 1977; Babkoff et al., 2002).

### Supra-threshold psychoacoustic tasks

#### Assessment of sensitivity to TE information

**Figure 6:**
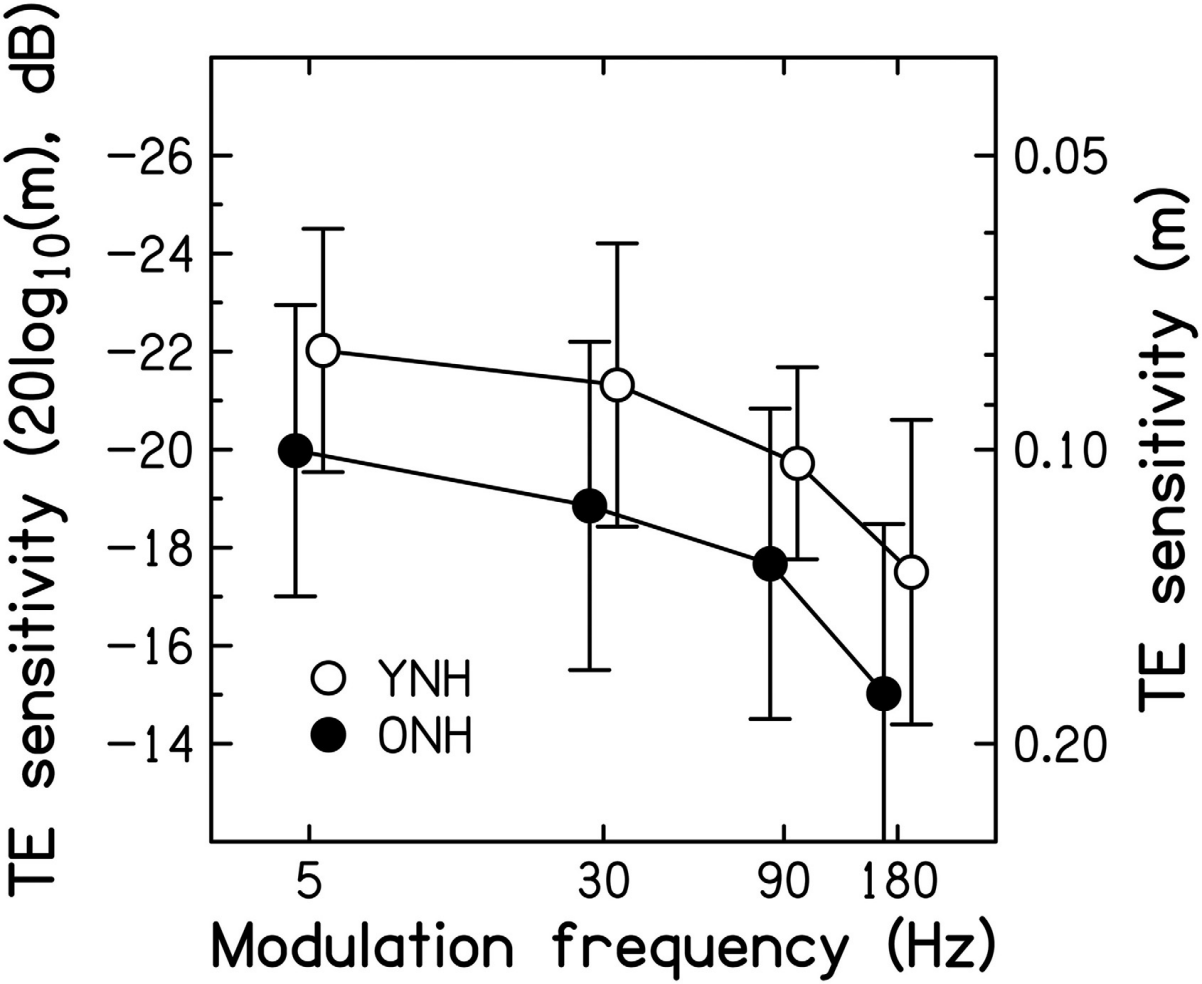
**Thresholds for detecting SAM, expressed as 20log_10_(*m*) in dB on the left axis, and as *m* on the right axis, as a function of modulation frequency in Hz**. Average thresholds for the YNH and ONH participants are indicated by the open and filled symbols, respectively. Error bars represent ±1 SD. Better sensitivity is toward the top of the figure.

Mean SAM detection thresholds for the two age groups^4^ are shown as a function of modulation frequency in Figure 6. To ease comparison with previously published results, the modulation depth at threshold (*m*, right axis) is expressed in dB, as 20log_10_(*m*), on the left axis. The TMTFs for both groups are similar in shape to those reported in previous studies for pure-tone carriers (Kohlrausch et al., 2000; Füllgrabe and Lorenzi, 2003).

On average, thresholds were 2–2.5 dB higher (worse) for the ONH than for the YNH participants. The effects of SAM frequency [*F*_(2.353, 61.190)_ = 20.132, *p* < 0.001] and Age group [*F*_(1, 26)_ = 4.208, *p* = 0.050] were significant, but the interaction was not [*F*_(2.353, 61.190)_ < 1, *p* < 0.946]. These results are generally consistent with previous studies reporting significant age-related decrements in the TMTF by 2–7 dB, as measured using pure-tone (He et al., 2008) and noise carriers (Takahashi and Bacon, 1992; Kumar and Sangamanatha, 2011), although in those studies the older participants had higher audiometric thresholds than the younger participants. Also, those studies showed the largest decrements for higher modulation frequencies, whereas here the decrement was independent of modulation frequency, suggestive of a deficit in processing efficiency and not temporal resolution (e.g., Hill et al., 2004). In other words, as for very young participants (i.e., normal-hearing children aged 4–7 years; Hall and IIIGrose, 1994), the peripheral encoding of TE information seems young-adult-like but the processing of this information is less efficient.

#### Assessment of sensitivity to TFS information

**Figure 7:**
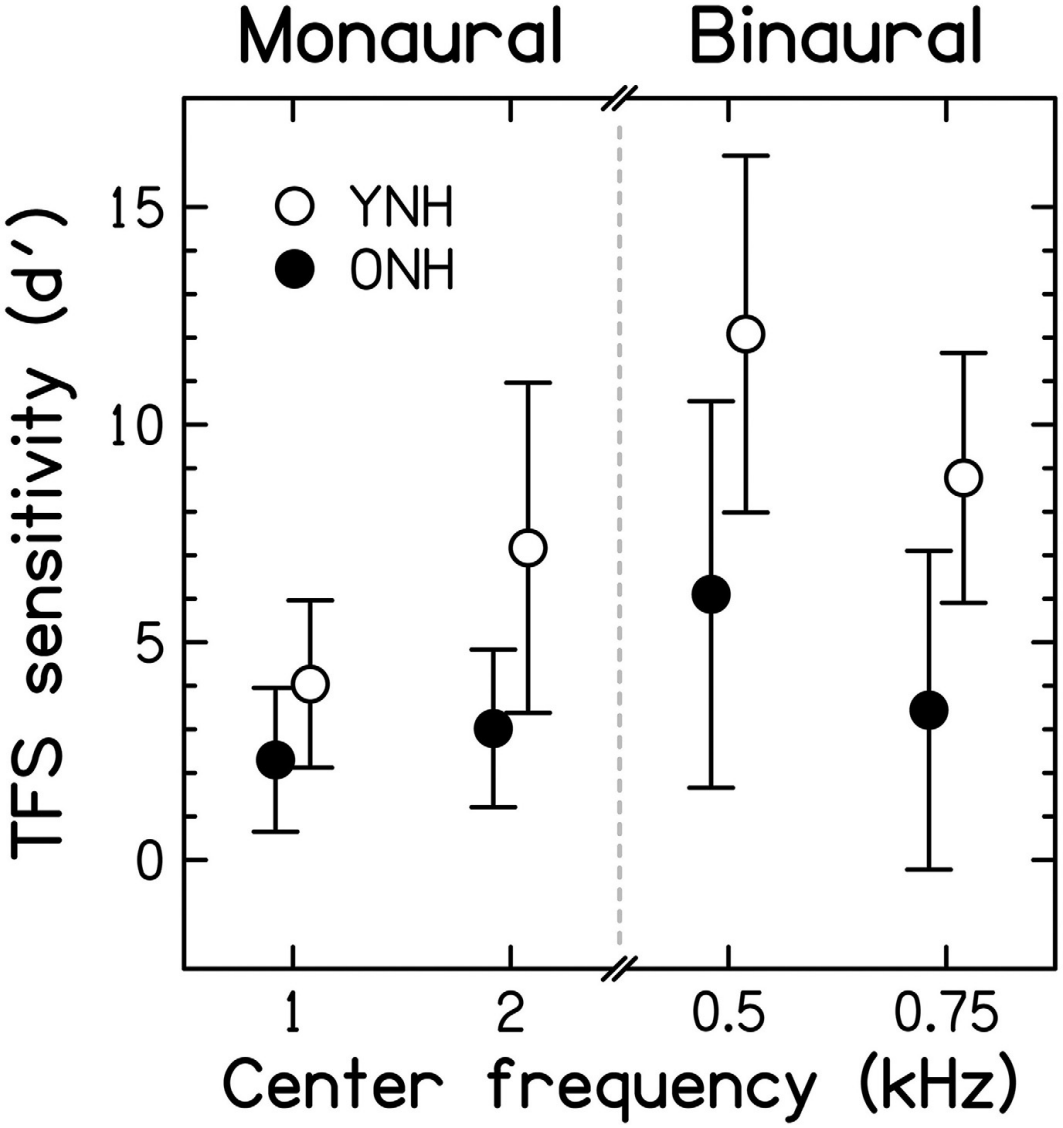
**Scores for TFS sensitivity, expressed in terms of the sensitivity index, *d*′, for the two fundamental frequencies used in the monaural TFS1 test and the two pure-tone frequencies used in the binaural TFS-LF test**. Open and filled symbols denote results for the YNH and ONH participants, respectively. Error bars represent ±1 SD. Better TFS sensitivity is toward the top of the figure.

To allow comparison of results from the adaptive and constant-stimulus procedures, thresholds (in Hz for the monaural test, and in degrees for the binaural test) and percent-correct scores were transformed into the value of the sensitivity index *d*' that would be obtained for the largest possible value of the manipulated variable, that is 0.5*F*0 for the TFS1 test and 180° for the TFS-LF test (for further details, see Hopkins and Moore, 2007, 2010). The *d*' values obtained in this way were sometimes very large. The utility of this conversion is that scores for both the adaptive and constant-stimulus procedures are transformed into a single scale, and values on this scale increase monotonically with improving performance. Each ear was tested separately in the TFS1 test. However, paired *t*-tests revealed no significant differences between the *d*' values for the left and right ears [*t*_(28)_ = −0.044, *p* = 0.965 and *t*_(28)_ = −0.747, *p* = 0.461 for the filter center frequencies of 1 and 2 kHz, respectively; both two-tailed]. Hence, results were pooled across the two ears for further analysis and presentation. Average *d*' values for the two age groups^5^ in the two monaural and the two binaural test conditions are shown in Figure 7.

Monaural TFS *d*' values for YNH participants were in good agreement with published data but *d*' values for the binaural TFS test were considerably higher (better) than previously observed (Moore and Sek, 2009; Hopkins and Moore, 2010, 2011; Moore et al., 2012b), possibly due to more protracted practice in the present study, to the longer tone duration, or to the longer interval between the two sets of four stimuli in each trial that was used here. Mean *d'* scores were higher for YNH than ONH participants. According to independent-samples *t*-tests, the differences between the age groups were significant [1 kHz: *t*_(27)_ = −2.427, *p* = 0.011; 2 kHz: *t*_(8.256)_ = −2.971, *p* = 0.009^6^; 0.5 kHz: *t*_(27)_ = −3.306, *p* = 0.002; 0.75 kHz: *t*_(27)_ = −3.703, *p* < 0.001; all one-tailed] and remained so after applying a Holm-Bonferroni correction for multiple comparisons. This confirms previous evidence for an age-related TFS processing deficit for smaller and/or audiometrically normal but unmatched participant groups (Hopkins and Moore, 2011; Moore et al., 2012b; Füllgrabe, 2013).

When measured at the same frequency (0.5, 1, or 2 kHz), audiometric thresholds (for each ear or averaged across the two ears) and TFS *d*' values (for each ear or for binaural processing) were not significantly correlated (*r* between 0.064 and −0.321; all *p* ≥ 0.090, uncorrected). Hence, TFS sensitivity for our normal-hearing participants was not associated with absolute threshold at the test frequency. Results from previous studies using audiometrically unmatched young and older (Hopkins and Moore, 2011; Moore et al., 2012b) or older participants with a range of ages (Moore et al., 2012a) generally agree with the present finding for binaural TFS sensitivity, but showed significant correlations between absolute threshold and monaural TFS sensitivity.

Surprisingly, the correlation between *d*' values for the two center frequencies used for the TFS1 test failed to reach significance (*r* = 0.322, *p* = 0.088), perhaps because individual differences in TFS sensitivity were relatively small at 1 kHz, or because TFS sensitivity might show idiosyncratic variations across frequency, even for audiometrically normal ears. However, *d*' values for the two center frequencies used for the TFS-LF test were highly correlated (*r* = 0.763; *p* < 0.001), and the correlation remained significant after partialling out the effect of age (*r*_−age_ = 0.663; *p* < 0.001). The *d*' value averaged over the two frequencies of the TFS1 test was moderately correlated with the *d*' value averaged over the two frequencies of the TFS-LF test (*r* = 0.541, *p* = 0.002), but the correlation became non-significant after partialling out the effect of age (*r* = 0.251, *p* = 0.197). This is consistent with previous suggestions that the TFS1 and TFS-LF tests tap partially different abilities (Hopkins and Moore, 2011; Moore et al., 2012b), perhaps because the latter involves additional binaural processing occurring in the brainstem.

### Cognitive tasks

**Figure 8:**
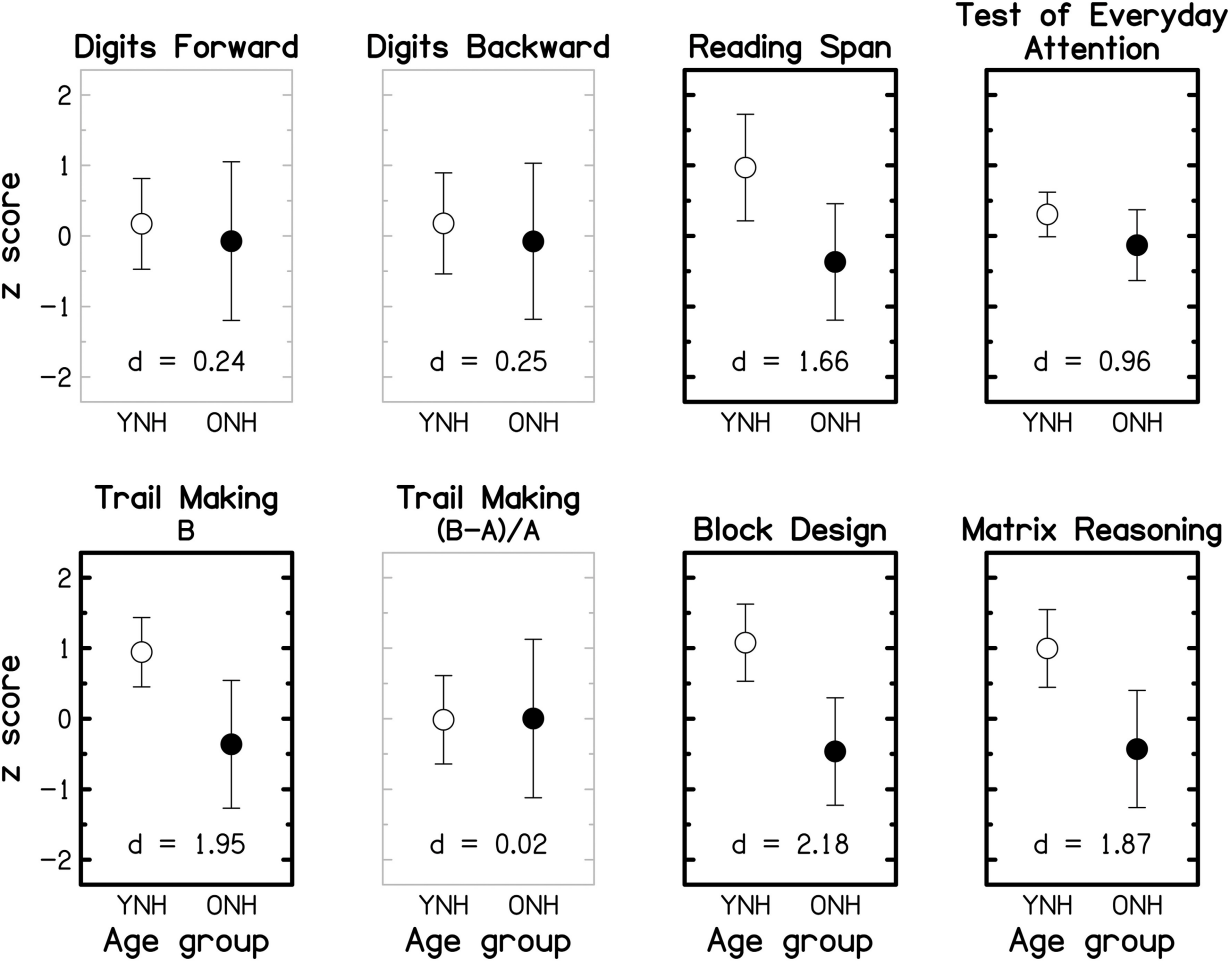
**Group-mean performance (in *z*-scores) for YNH (open symbols) and ONH (filled symbols) participants on different cognitive tasks**. Error bars represent ±1 SD. Gray panel frames indicate non-significant group differences (*p* > 0.05). Bold black panel frames denote significant results at *p* ≤ 0.05 that remained significant after applying a Holm-Bonferroni correction. The effect size is given by Cohen's *d* at the bottom of each panel.

To facilitate comparison across cognitive tests and with findings of previous cognitive-aging studies (e.g., Park et al., 2002; Salthouse, 2009), the data were transformed into *z*-scores, using the mean and the SD of the entire group (YNH and ONH combined), prior to statistical analyses. Reaction-time data were multiplied by -1 after being transformed into *z*-scores so that better performance was represented by higher *z*-scores across all tests. Group means and SDs for the seven tests (plus the derived measure for the TM test) are shown in Figure 8 for the YNH (open symbols) and OHN (filled symbols) participants. Performance for the TEA was computed as the average of the unit-weighted *z*-scores for the eight sub-tests. For each cognitive measure, the effect size, expressed as Cohen's *d* ^7^, is given at the bottom of the panel. Gray and black panel frames denote non-significant (*p* > 0.05) and significant (*p* ≤ 0.05) group differences, respectively. Bold panel frames indicate differences that remain significant after applying a Holm-Bonferroni correction.

For all tests, mean scores were higher for the YNH than for the ONH participants, but the effect size varied from small (*d* ~ 0.2) for the two DS tests, to large (*d* >~ 0.8) for the remaining tests. Performance on each of the two DS tests and the derived measure for the TM test did not differ significantly for the two age groups (all *p* ≥ 0.461; two-tailed; uncorrected), but all other tests showed significant effects of age group (all *p* ≤ 0.011; two-tailed; uncorrected) which remained significant after correcting for multiple comparisons. The group means of the raw scores for the eight cognitive measures and the results of independent-samples *t*-tests are given in the Supplementary Material: *Raw scores and statistical results for cognitive measures*.

It is often assumed that the DS-B and RS require both information storage and processing, while the DS-F involves only information storage. However, performance on the RS test, but not on the two DS tests, was significantly affected by age, suggesting that the “re-ordering task” (DS-B) is more closely related to STM tests (such as DS-F) than to complex WM tests (for a discussion of this point, see Bopp and Verhaeghen, 2005). This interpretation is supported by a significant correlation between scores for the two DS tests (*r* = 0.622, *p* < 0.001; two-tailed) but non-significant correlations between scores for either of these tests and RS scores (both *r* ≤ 0.271, both *p* ≥ 0.155; two-tailed).

**Figure 9:**
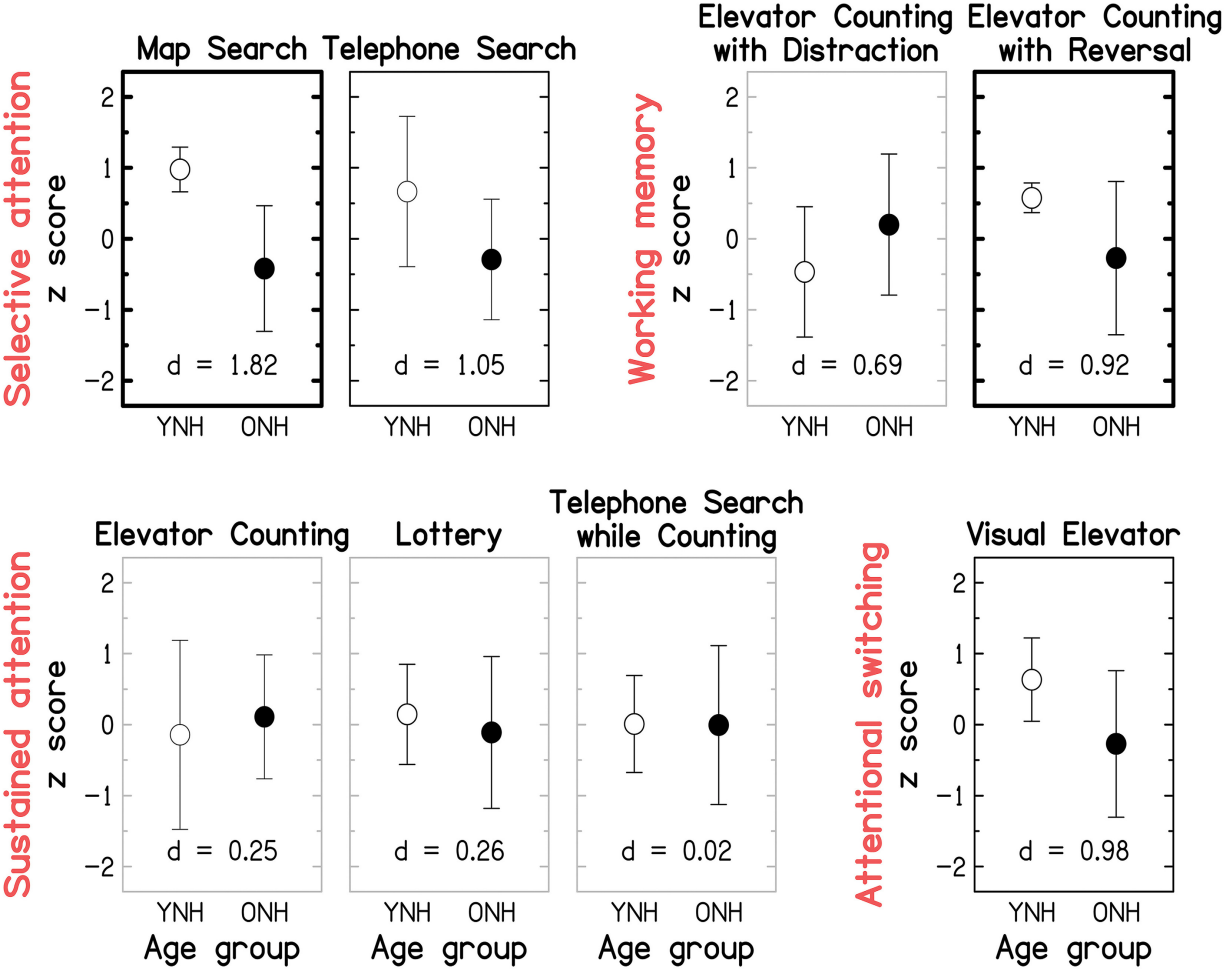
**Group-mean performance (in *z*-scores) for YNH (open symbols) and ONH (filled symbols) participants on each of the eight sub-tests of the Test of Everyday Attention**. Sub-tests are grouped by the underlying attentional processes (see red labels) they are assumed to assess according to Robertson et al. (1996): Selective attention (Map Search, Telephone Search), Audio-verbal working memory (Elevator Counting with Distraction, Elevator Counting with Reversal), Sustained attention (Elevator Counting, Lottery, Telephone Search while Counting), and Attentional switching (Visual Elevator). Gray panel frames indicate non-significant group differences (*p* > 0.05). Black panel frames denote significant results at *p* ≤ 0.05. Bold panel frames indicate significant results after applying a Holm-Bonferroni correction. Otherwise as Figure 8.

Figure 9 gives the scores for each of the eight sub-tests of the TEA, grouped by the putatively assessed attentional process identified by (Robertson et al., 1996); note that subsequent factor analyses only partially confirmed these groupings (Chan, 2000; Bate et al., 2001). Effect size and statistical significance are indicated for each sub-test, as for Figure 8. The raw mean scores and statistical results are given in the Supplementary Material: *Raw scores and statistical results for cognitive measures*. The pattern of results is broadly consistent with the nomenclature (see red labels in Figure 9) suggested by Robertson et al. (1996): large and significant age effects (all *p* ≤ 0.021; two-tailed; uncorrected) were observed for both of the selective-attention tests, one of the WM tests, and the attentional-switching test, although the effects became non-significant for the Telephone Search and Visual Elevator tests after correction for multiple comparisons. All three tests of sustained attention yielded small and non-significant age effects (all *p* ≥ 0.521).

Given that the TEA was designed as a neuropsychological screening tool, it is not surprising that ceiling effects were observed for some of the sub-tests (Robertson et al., 1996). In our “healthy” sample, most participants performed perfectly on the Elevator Counting test and many YNH participants scored close to ceiling on the Map Search test. At least for the latter test, administering version B of the test might overcome this problem in the future; indeed, a group of 31 YNH participants tested on that version as part of an unrelated study yielded a lower mean score of 65.3/80 (compared to 75.4/80 in the current study).

### Correlation and regression analyses

The strength of the association between supra-threshold auditory processing, various cognitive abilities, and SiN identification was evaluated by conducting correlation and regression analyses. However, the analysis of data from “extreme” age groups using these statistical tools can be problematic (Hofer et al., 2003). As demonstrated in the previous section, TE and TFS sensitivity, cognitive processing, and speech perception were all generally poorer for the ONH than for the YNH group. Even if no association between psychoacoustic, cognitive, and speech measures existed within each age group, use of the combined scores across all participants could reveal a significant relationship between the measures. To avoid this pitfall, we followed the example of Grassi and Borella (2013), and computed correlations not only across all participants, but also restricting the analyses to the data for the ONH group, and also after partialling out the effect of age.

#### Relationship between auditory temporal processing and speech perception

To reduce the effect of errors of measurement, masked speech-identification scores for each participant were averaged across the different SNRs and masker types, to give a single composite score for consonants and a single composite score for sentences. Similarly, a composite score for TE sensitivity was obtained by averaging detection thresholds for the four modulation frequencies, and a composite score for TFS sensitivity was obtained by averaging *d*' values across the two TFS1 and the two TFS-LF conditions.

**Figure 10:**
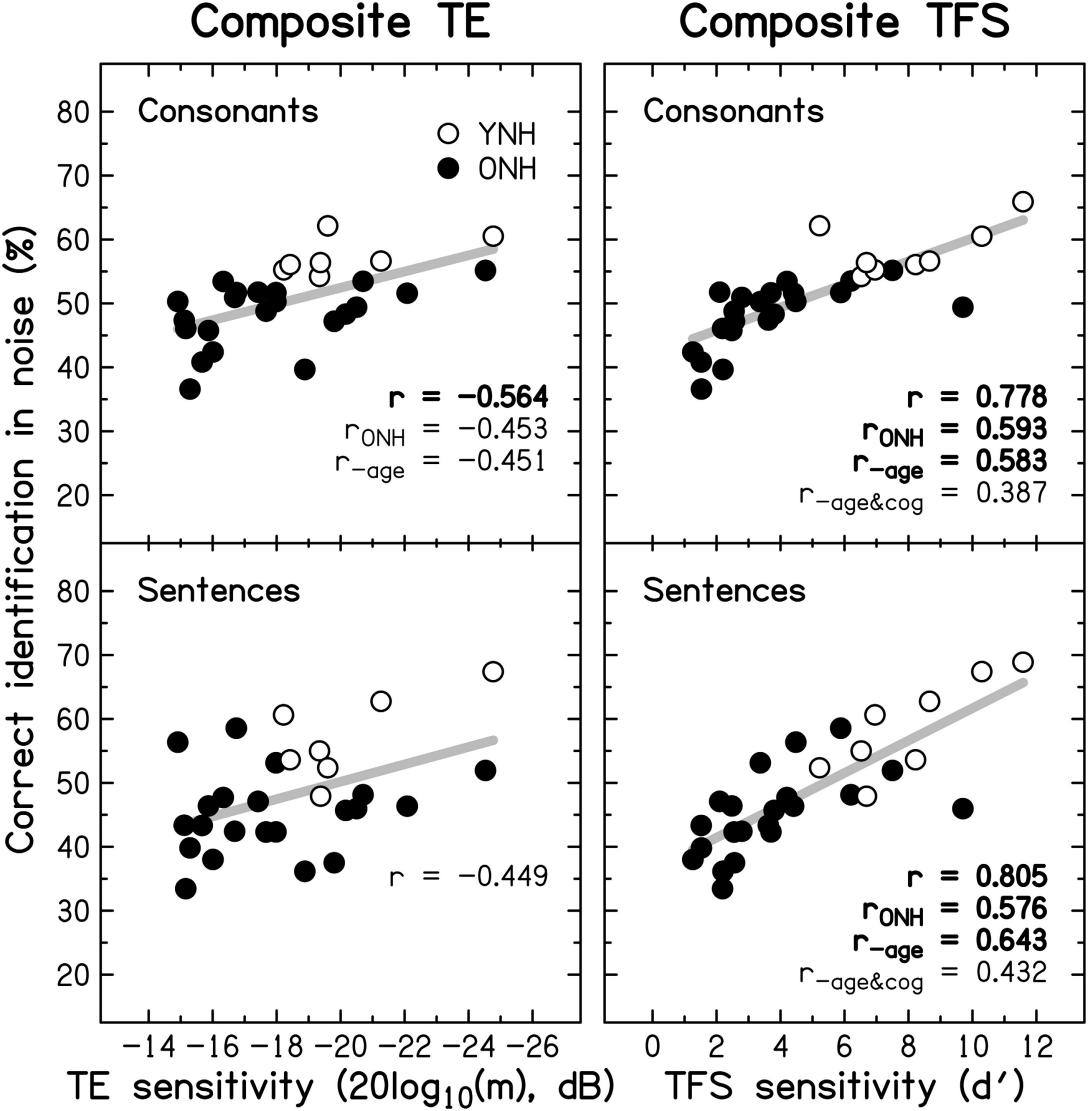
**Scatter plots of composite sensitivity to TE (left panel) and TFS (right panel) vs. composite consonant (top row) and sentence (bottom row) identification in noise**. The thick gray line represents the best linear fit to the data from the entire group composed of YNH (open symbols) and ONH participants (filled symbols). Significant (at *p* ≤ 0.05; uncorrected) correlation coefficients for all participants (*r*), for the ONH participants only (*r*_ONH_), and for all participants with age (*r*_−age_) or with age and composite cognition (*r*_−age&cog_) partialled out, are given in each panel. Bold font indicates significance at *p* ≤ 0.001.

Figure 10 shows individual composite consonant and sentence identification scores for the YNH and ONH participants plotted against the composite measures of TE sensitivity (left column) and TFS sensitivity (right column). In each panel, significant correlation coefficients are given (*r* for the entire group, *r*_ONH_ for the ONH group only, and *r*_−age_ for the entire group when age was partialled out). The boldness of the font increases with increasing significance (from *p* ≤ 0.05 to 0.001). For the entire group, speech scores were strongly and significantly associated with TFS sensitivity, and were somewhat more weakly associated with TE sensitivity. When only the ONH group was considered, or participant age (alone or together with composite cognition; see Section *Relationship between cognitive abilities and speech perception*) was partialled out, the strength of the correlation between TFS sensitivity and performance on both speech tasks was somewhat reduced but remained significant. In contrast, TE sensitivity was no longer significantly associated with sentence identification and its correlation with consonant identification, while still significant at *p* ≤ 0.05, was only moderate.

Given the small number of YNH participants in this study no detailed correlational analysis for this group is presented. However, it is noteworthy that for our YNH sample TFS sensitivity was correlated strongly with sentence identification in noise (*r* = 0.839, *p* = 0.009). Neher et al. (2011) did not find a correlation between TFS sensitivity and a measure of speech perception for a similarly sized “youngish” normal-hearing group. However, they only assessed the relationship for binaural TFS sensitivity and for target speech presented at a different azimuth from the speech maskers.

**Figure 11:**
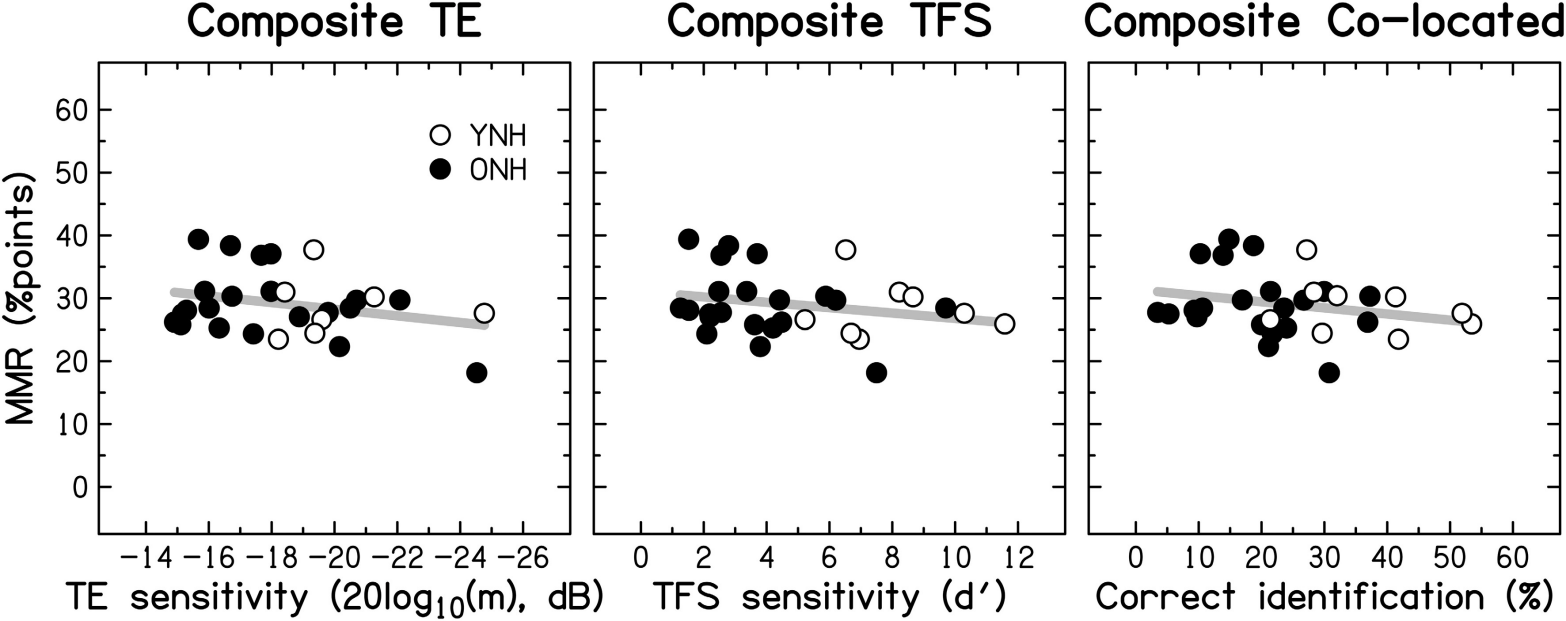
**Scatter plots of MMR for consonant identification vs. composite sensitivity to TE (left panel), composite sensitivity to TFS (middle panel), and composite sentence identification in the presence of co-located two-talker babble (right panel)**. Otherwise as Figure 10.

Based on the evidence that sensorineural hearing loss is associated with a reduced ability to process TFS information (e.g., Buss et al., 2004; Lacher-Fougère and Demany, 2005; Santurette and Dau, 2007; for an overview, see Moore, 2014), some authors (Lorenzi and Moore, 2008; Moore, 2008; Hopkins and Moore, 2009) have suggested that the large speech-perception deficit experienced by hearing-impaired listeners in the presence of modulated noise could be a consequence of their inability to use TFS information to take advantage of the minima in the noise. Similarly, it is often assumed that dip listening requires a certain degree of temporal resolution (Festen, 1993; Stuart and Phillips, 1996; Füllgrabe et al., 2006; George et al., 2006; Grose et al., 2009). To test the role of TE and TFS sensitivity in MMR, and its dependence on age, a composite measure of MMR was calculated for the consonant-identification task, by averaging individual scores across the different SNRs and two SAM frequencies. The scatter plots in Figure 11 indicate that MMR was not significantly associated with the composite measures of TE sensitivity (left panel; *r* = 0.280, *p* = 0.148) or TFS sensitivity (middle panel; *r* = −0.233, *p* = 0.224). Also, MMR was not significantly correlated with composite sentence identification in the presence of co-located speech interference (right panel; *r* = −0.204, *p* = 0.279).

#### Relationship between cognitive abilities and speech perception

The association between cognitive measures and identification of consonants and sentences in noise was assessed for the ONH group and for the entire group after partialling out the effect of age (see Table 1). Correlation coefficients significant at *p* < 0.05 are shown in black.

**Table 1:**
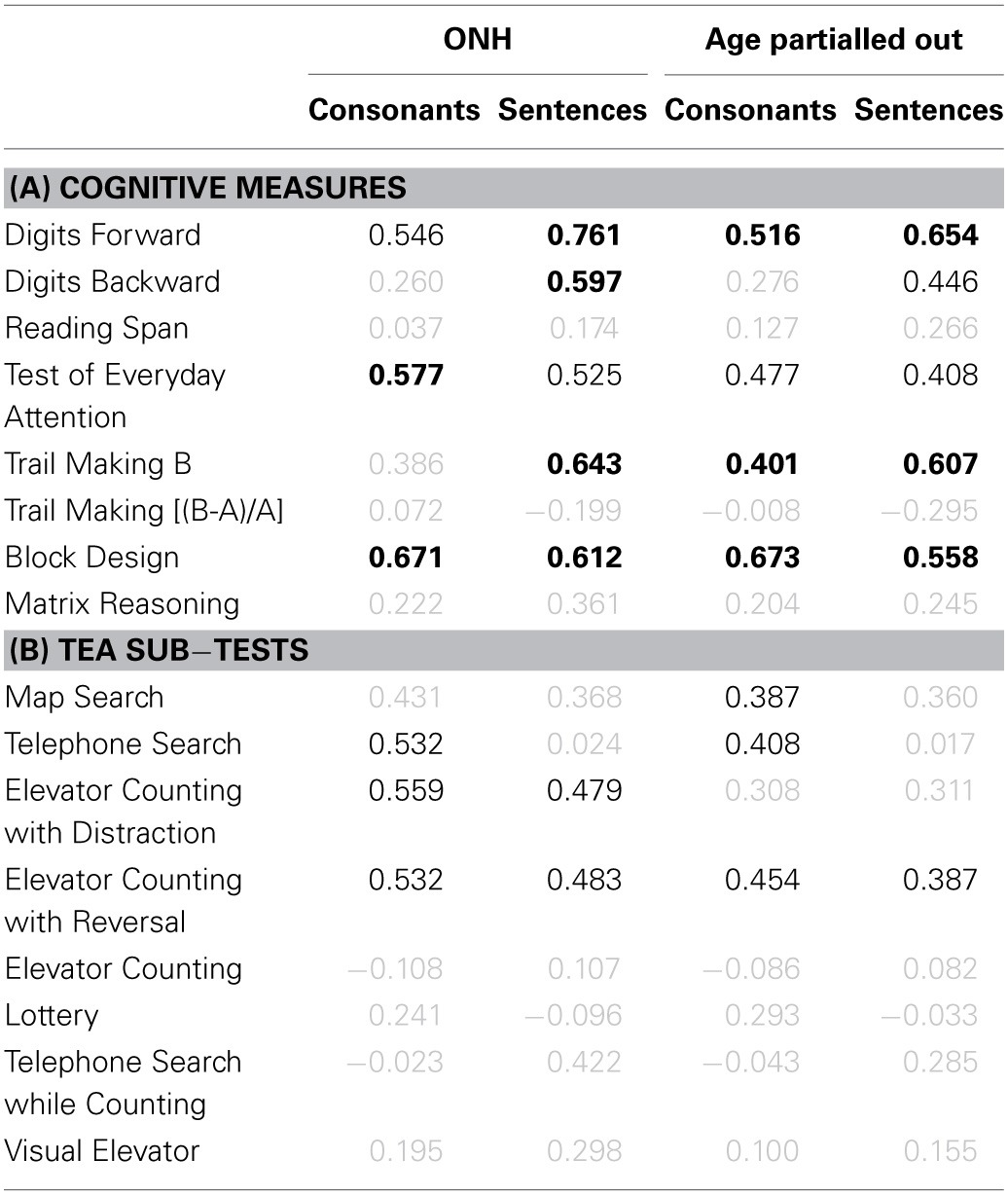
**(A) Pearson product-moment correlation coefficients for results on eight cognitive measures vs. consonant- (first and third result columns) and sentence-identification performance in noise (second and fourth result columns). Results for the ONH group only and for the entire group after partialling out participant age are given in result columns 1–2 and 3–4, respectively. Gray values indicate non-significant correlations (*p* > 0.05). Values in black indicate significant results at *p* ≤ 0.05. Values in boldface indicate significant results after applying a Holm-Bonferroni correction. (B) Correlation coefficients for performance on the eight sub-tests of the TEA vs. speech-identification performance in noise. Otherwise as (A)**.

Somewhat similar patterns of results were observed for the two correlational analyses for the two speech tasks. Considering only those results that remained significant after applying a Holm-Bonferroni correction (values in boldface), mainly scores for DS-F, DS-B, TM-B, and BD were correlated consistently with speech identification scores. Performance on the RS test was not significantly associated with speech perception (even though there was a significant moderate and positive correlation when young and older participants were considered together, consistent with results reported by Besser et al., 2012). This finding contrasts with a growing body of evidence that WM capacity, as measured by the RS test, is correlated with speech perception for hearing-impaired listeners (e.g., Rudner et al., 2011), and does not support the notion that “WM capacity also seems to play an important role when people with normal hearing must understand language spoken in acoustically adverse conditions” (Rönnberg et al., 2013). A survey of previous studies administering the RS test and a measure of SiN perception to YNH participants revealed a mixed pattern of results: while Moradi et al. (2014) reported a significant (but uncorrected) moderate positive correlation between scores on the two tasks, others either found significant results only for a sub-set of the tested SiN conditions (Zekveld et al., 2011; Besser et al., 2012; Kilman et al., 2014) which, contrary to predictions, did not always include the most adverse conditions, or failed to find any evidence for a relationship between WM capacity and SiN performance (Zekveld et al., 2014). The significant correlations (but uncorrected for multiple comparisons) found in studies including adults from a wider age range with no or only partial audiometric confirmation of normal hearing were possibly confounded by age-related changes in audibility, supra-threshold auditory processing, and/or cognition (Besser et al., 2012; Ellis and Munro, 2013). Consistent with this, Besser et al. (2012) reported that the moderate correlation between performance on the RS and speech test was no longer significant after partialling out the effect of age. In summary, it is currently unclear if individual differences in WM capacity in the audiometrically normal-hearing young or older population are the main contributor to the observed variability in SiN perception. Further studies are warranted to explicitly address this issue, including the questions: (1) is the RS test the most appropriate measure of WM (Besser et al., 2012; Sörqvist and Rönnberg, 2012); (2) which of the sub-processes of WM does the RS test probe (Unsworth and Engle, 2007; Sörqvist et al., 2010); and (3) what constitutes an acoustically adverse condition?

**Figure 12:**
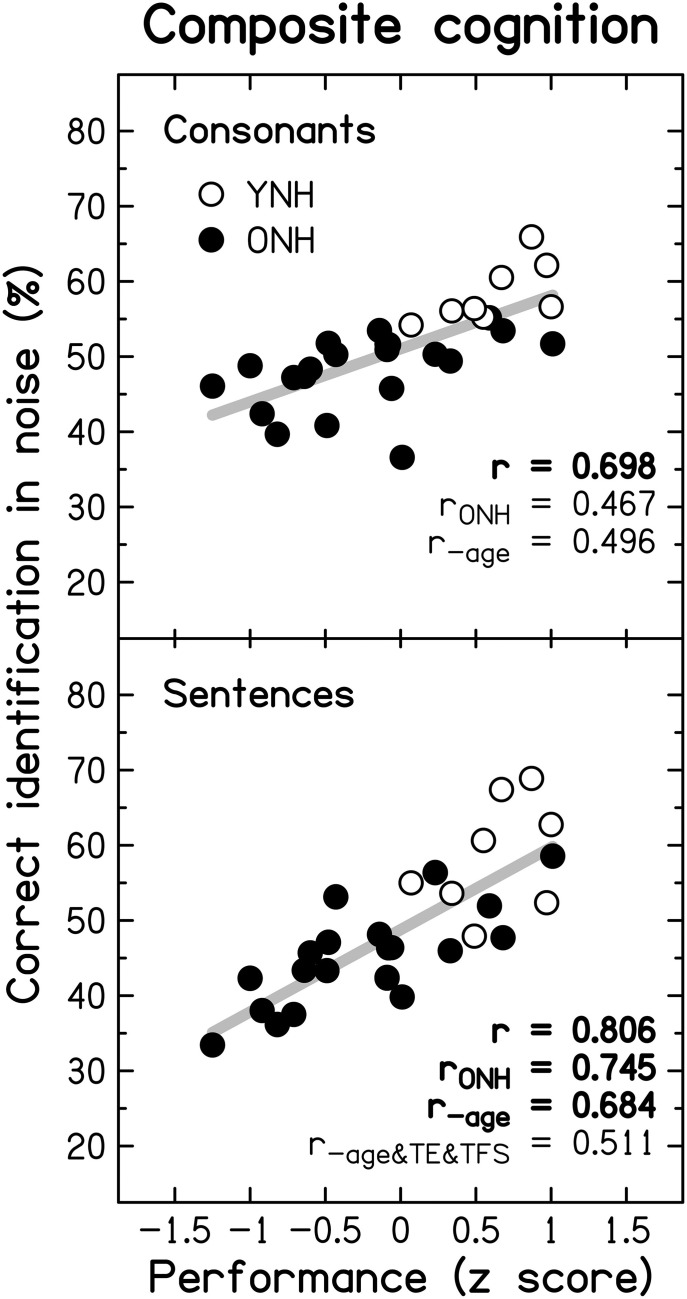
**Scatter plots of composite cognition vs. consonant (top row) and sentence identification in noise (bottom row)**. Significant (at *p* ≤ 0.05; uncorrected) correlation coefficients for all participants (*r*), for the ONH participants only (*r*_ONH_), and for all participants with age (*r*_−age_) or with age and composite TE and TFS sensitivity (*r*_−age&TE&TFS_) partialled out, are given in each panel. Otherwise as Figure 10.

In an attempt to characterize the relationship between general cognitive functioning and speech perception, a composite cognition score was computed by averaging the unit-weighted *z-scores* (Salthouse, 1991; Lindenberger et al., 2001) from all eight cognitive measures, independently of whether or not they were associated with speech perception. Such an all-inclusive approach was meant to avoid “cherry picking” the cognitive tests yielding the strongest correlations with speech perception. Figure 12 shows the scatter plots of scores for identification of consonants and sentences in noise against the composite cognition scores.

Considering the entire participant group, scores for both speech tasks were strongly associated with cognition. Limiting the analysis to the ONH group, or partialling out the effect of age, reduced the strength of the correlation but it remained moderate (for consonant identification) to strong (for sentence identification). All analyses yielded larger correlation coefficients and smaller *p* values for sentence than for consonant identification. Interestingly, the correlation between cognition and sentence identification was still moderate and significant (*p* = 0.009) after partialling out the effects of age, composite TE sensitivity, and composite TFS sensitivity.

Despite the link of cognition with consonant identification, neither form of release from masking was associated with cognitive functioning: MMR, *r* = −0.198, *p* = 0.303; SMR, *r* = −0.125, *p* = 0.519. In other words, the ability to benefit from temporal dips in a masker or spatial separation between a target and masker was not related to cognition.

#### Relationship between cognitive abilities and temporal auditory processing

Since a link between cognitive abilities (especially WM capacity) and temporal processing has recently been suggested (Troche and Rammsayer, 2009; Broadway and Engle, 2011), correlations were computed between composite sensitivity scores for TE and TFS and the eight cognitive measures (see Table 2A), and the eight sub-tests of the TEA (see Table 2B). No evidence was found that performance on the RS test, assumed to index WM capacity, was linked to temporal processing abilities. However, for several other cognitive tests (DS-F, BD, TEA) there were significant positive moderate correlations, mainly with TFS sensitivity, even when the effect of age was partialled out. Amongst the TEA sub-tests, scores for one selective-attention test (Map Search) and the two WM tests (Elevator Counting with Distraction and Elevator Counting with Reversal) were significantly correlated with TFS sensitivity. The relationship between cognition and TFS processing might occur because some level of cognitive ability is required to perform well on the TFS tests. Alternatively, or in addition, it may occur because both are linked to the integrity and precision of neural processing.

**Table 2:**
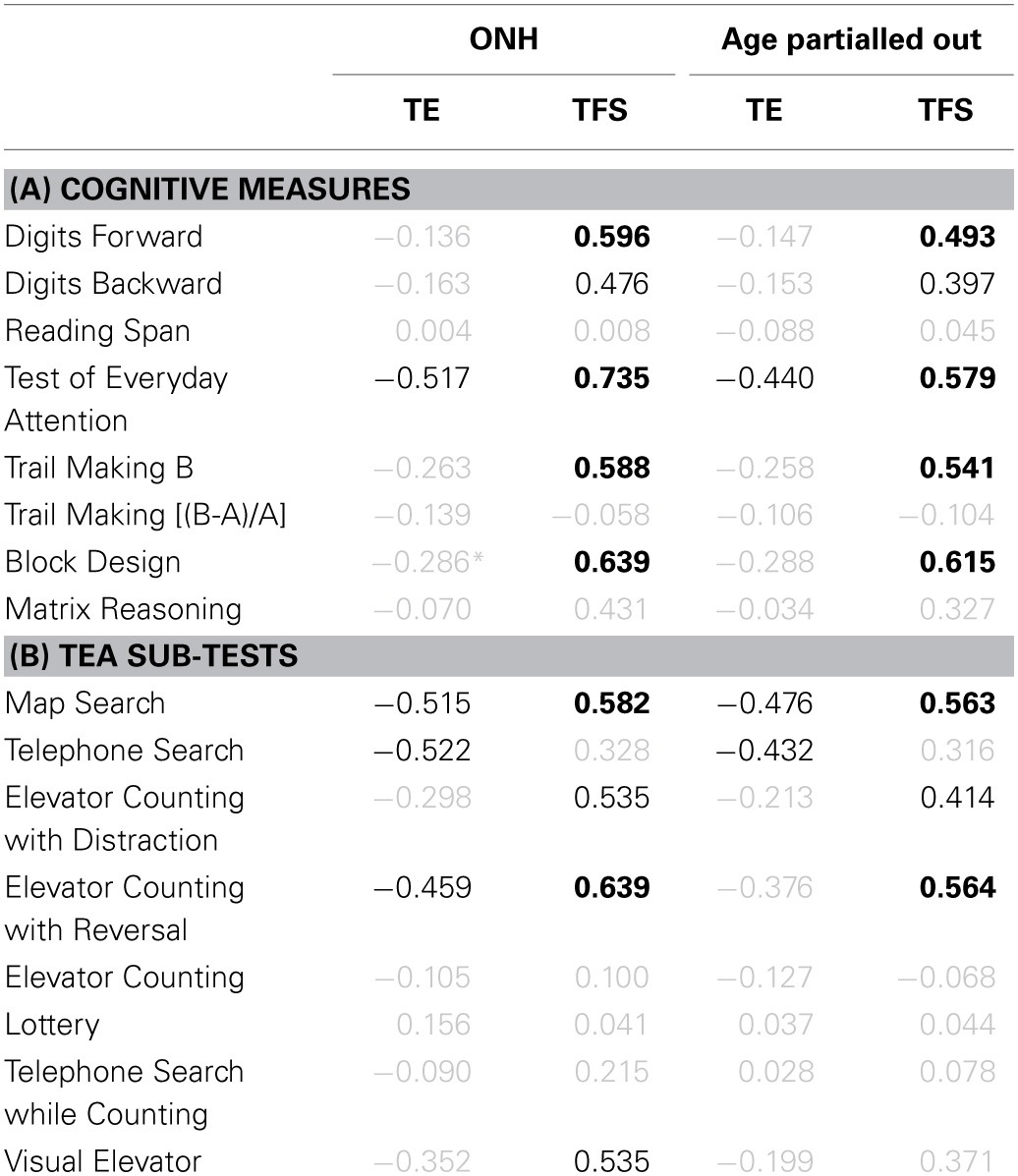
**(A) Pearson product-moment correlation coefficients for results on eight cognitive measures vs. composite TE (first and third results columns) and composite TFS sensitivity (second and forth result columns). Otherwise as Table 1. (B) Correlation coefficients for performance on the eight sub-tests of the TEA vs. composite temporal sensitivity. Otherwise as (A)**.

#### Multiple regression analysis

To explore the relative importance of the factors contributing to the variance in consonant and sentence identification, multiple regression analyses (using the stepwise method) were carried out separately for the two speech tasks, using composite scores for TE sensitivity, TFS sensitivity, and cognition as predictor variables.

For consonant identification, the most parsimonious significant model that emerged was based on the single predictor TFS sensitivity [*F*_(1, 26)_ = 28.826, *p* < 0.001]. The model explained 50.8% of the variance. The standardized regression coefficient for the TFS variable was 0.725 (*p* < 0.001).

For sentence identification, the significant model that explained the most (68%) of the variance (adjusted *R*^2^ = 0.68) was based on Cognition and TFS sensitivity [*F*_(2, 25)_ = 29.679, *p* < 0.001]. The standardized regression coefficients were 0.509 (*p* = 0.004) for cognition and 0.392 (*p* = 0.020) for TFS sensitivity.

## Summary and general discussion

Increasing acknowledgement of speech identification and comprehension problems among older people (for a review, see Gordon-Salant et al., 2010), combined with awareness of the increasing proportion of older people in most Western countries (e.g., Christensen et al., 2009), has spawned a considerable number of studies investigating the age-related auditory and cognitive changes that underlie speech perception problems. In the absence of gross cognitive dysfunction, elevated audiometric thresholds in older listeners have been identified as the major contributor to the reduction in speech intelligibility (Van Rooij and Plomp, 1992; Humes, 1996, 2007; Dubno et al., 2008). However, audibility generally did not explain all of the variance in identification performance.

### Study aims, findings, and implications

The aims of the present study were to confirm the existence of SiN identification difficulties in the older population and to investigate the nature and relative importance of the associated age-related changes in supra-threshold auditory and cognitive processing. The following steps were taken to control for the roles of audibility and cochlear status in SiN identification: (1) only young and older participants with normal audiograms over a wide frequency range (0.125–6 kHz) were included; (2) the average audiograms of the two groups were matched; and (3) high-frequency information from the signals was removed to ensure zero audibility of frequency components above 6 kHz for both age groups. Two speech tasks were administered to capture different levels of processing and complexity: consonant identification in unmodulated or modulated speech-shaped noise under “dry” conditions, and sentence identification in spatially co-located or separate speech maskers under reverberant conditions. To clarify the factors contributing to individual and age-group performance on these tasks, participants were also characterized in terms of: (1) their sensitivity to TE and TFS cues, which are known to be important for speech intelligibility and auditory scene analysis; and (2) their performance on a battery of cognitive tests probing cognitive abilities such as memory, attention, and processing speed. The main findings are summarized below:

1. Identification scores for consonants in quiet and in speech-shaped noise were lower for ONH than for YNH participants. The size of this effect was independent of masker type (unmodulated vs. modulated), masker modulation frequency (5 vs. 80 Hz), and SNR.
2. Modulation masking release did not differ for the two age groups.
3. Identification scores for sentences in quiet were identical for the two age groups, but identification of sentences in the presence of spatially co-located or separate interfering two-talker babble was worse for the ONH group. The size of this age effect was similar across SNRs within the same masker-location condition for SNRs at which performance was not affected by a floor or ceiling effect.
4. Spatial masking release did not differ for the two age groups.
5. The lower speech-perception performance of the ONH participants was not associated with higher subjective ratings of hearing disabilities.
6. Sensitivity to TE information was reduced for ONH participants; modulation detection thresholds were higher by 2–2.5 dB across all modulation frequencies used (*f_m_* = 5–180 Hz).
7. Sensitivity to monaural and binaural TFS information was reduced for ONH participants.
8. Performance on most, but not all, cognitive tests was worse for the ONH than for the YNH participants. Cognitive abilities spared by aging included short-term memory and sustained attention.
9. Composite sensitivity to temporal information was correlated positively and moderately (for TE sensitivity) or strongly (for TFS sensitivity) with consonant and/or sentence identification in noise. After partialling out the effects of age and composite cognition, a moderate correlation with TFS sensitivity remained, and was significant.
10. Composite sensitivity to TE or TFS was not correlated significantly with modulation masking release. Neither was sentence identification in co-located two-talker babble.
11. Performance on some cognitive tests was correlated positively and moderately to strongly with TFS sensitivity. Correlations were moderate, but remained significant, after partialling out the effect of age.
12. The measure of composite cognition was correlated positively and strongly with consonant and sentence identification in noise. After partialling out the effects of age, TE sensitivity, and TFS sensitivity, a moderate correlation with sentence identification remained, and was significant.
13. Composite cognition and sensitivity to TFS explained 68% of the variance in sentence-in-speech identification. Composite TFS sensitivity explained 51% of the variance in consonant-in-noise identification.

Most of the age-group differences in auditory and cognitive processing and their associations with intelligibility in noise were statistically significant. However, despite these deficits in test performance, the ONH participants did not report more hearing disabilities than the YNH participants on either of the two questionnaires used (for possible explanations, see the Supplementary Material: *Discrepancy between measured and self-assessed hearing difficulties*). Thus, the practical significance of these age effects for speech processing in everyday life remains to be determined. It is likely that age-related deficits are even more pronounced in more variable listening conditions (for effects of stimulus variability, see Sommers, 1997; Golomb et al., 2007) and when the speech material has greater syntactic complexity (Wingfield et al., 2006) than used here, even when compensatory mechanisms (Wingfield and Grossman, 2006; Reuter-Lorenz and Cappell, 2008) and changes in cognitive strategies (Lemaire, 2010) might help to offset the deleterious effects of variability and complexity (e.g., through the enhanced use of contextual knowledge; Pichora-Fuller et al., 1995; Wingfield, 1996; Dubno et al., 2000; Pichora-Fuller, 2008). Also, the impact of aging on speech communication might manifest itself in other ways than in a decrement in identification performance (e.g., changes in conversational discourse pragmatics; Kiessling et al., 2003; McKellin et al., 2007).

In a recent review of the literature on the topic of age-related central factors in presbyacusis, Humes et al. (2012) concluded that, given the lack of control of confounding variables such as hearing sensitivity and cognition in most studies, there is insufficient evidence to support a “pure” form of central presbyacusis (i.e., age-related central auditory decline in the absence of peripheral and/or cognitive changes). Our data showed a significant negative correlation between age and composite TFS (but not TE) sensitivity in our audiometrically and performance-IQ-matched normal-hearing participants, even after partialling out the effect of composite cognition (*r*_−cog_ = −0.450, *p* = 0.016; two-tailed). Also, the correlation between age and the identification of meaningless VCVs in noise (a condition in which participants mainly had to rely on acoustic cues) was significant, even after partialling out the effect of composite cognition (*r*_−cog_ = −0.475, *p* = 0.011; two-tailed)^8^. These findings support the idea of a form of presbyacusis that is not confounded by age-related cognitive changes and that is not related to changes in the cochlea that lead to elevated audiometric thresholds. The presbyacusis could be a result of neural changes anywhere from the auditory nerve up to higher centers in the auditory system (e.g., Sergeyenko et al., 2013).

Despite an increasing body of evidence showing age deficits affecting various aspects and levels of auditory and cognitive processing, many experimental studies investigating the perceptual consequences of hearing loss on auditory perception did not use age-matched experimental groups, but compared *young* normal-hearing to *older* hearing-impaired participants (for an example from previous work by the authors, see the Supplementary Material: *Confounding age effect in a study of hearing loss*). Consequently, it is likely that many published results overestimate the effects of hearing loss as measured by the audiogram, and need to be “corrected” for the effect of age. A similar word of caution applies to many studies of aging, in which audibility across age groups was generally not matched and often only loosely controlled for, in spite of clear evidence that differences in audiometric thresholds can result in differences in speech identification (Humes, 1996; Dubno and Ahlstrom, 1997; Demeester, 2011).

### Future directions

The use of the audiogram as the main clinical measure of hearing status, and the use of the diagnostic term presbyacusis (literally “elderly hearing”) to refer to “*age*-related *hearing* loss” both reflect the common assumption that the speech processing difficulties of older persons are mainly related to, and are predictable from, their audiogram. However, the data reported here confirm and extend evidence that has been accumulating over several decades, showing that, even when the audiogram is normal, deficits in central-auditory and cognitive processing are ubiquitous in the older population and are associated with poorer speech identification. This highlights the need to expand audiological assessment beyond tests of pure-tone audibility (Kricos, 2006) and to devise effective rehabilitative interventions for speech-perception difficulties in older listeners that target not only peripheral dysfunction through frequency-specific amplification *via* hearing aids but also age-related changes in central auditory and cognitive functions through the provision of auditory-based perceptual training programs (Dubno, 2013; Ferguson et al., 2014), targeted cognitive-strategy and cognitive-process training regimens (Lustig et al., 2009; Park and Bischof, 2013), and general cognitive enrichment (Hertzog et al., 2008).

The use of an extreme-group cross-sectional approach in most aging studies precludes the possibility of determining the time of onset of changes within the adult auditory and cognitive systems. Several recent studies have used an intermediate age group to examine whether age-related deficits are already present in midlife (e.g., Ross et al., 2007; Schvartz et al., 2008; Humes et al., 2013a; Helfer and Freyman, 2014). More accurate estimates of when the first signs of aging in auditory and cognitive performance become apparent can be derived from cross-sectional studies sampling continuously across the entire adult life span (Bergman et al., 1976; Baltes and Lindenberger, 1997; Park et al., 2002; Salthouse, 2009; Füllgrabe, 2013) or longitudinal studies (Dubno et al., 2008; Payne et al., 2014). It is also those studies that will inform us about the shape of the trajectory of the decline throughout adulthood.

## Conclusions

Taken together, the results show that, even in the absence of hearing loss as measured by the audiogram, SiN identification declines with age. Both consonant and sentence identification were poorer for the older participants, but possibly not for the same reasons. For both speech tasks, sensitivity to TFS information, which is thought to facilitate the parsing of auditory scenes into sound sources, was more important than sensitivity to TE information. When the target speech consisted of meaningful utterances presented against a background of interfering speech, identification performance was best predicted by cognitive abilities and, to a lesser extent, sensitivity to TFS information. Neither MMR nor SMR differed across age groups. These findings indicate a need for clinical tests in addition to the audiogram when assessing the hearing of older people, and confirm the need to take age into account in studies examining the effects of hearing loss.

## Conflict of interest statement

The authors declare that the research was conducted in the absence of any commercial or financial relationships that could be construed as a potential conflict of interest.

## Footnotes

^1^Here and for subsequent ANOVAs, when the assumption of sphericity was not met, the Greenhouse-Geisser correction was used.

^2^We deliberately refrain from using the qualifiers “steady” or “steady-state” that are generally used in the literature to refer to a noise on which no amplitude modulation is impressed. Recent work by Stone et al. (2011, 2012) indicates that the random amplitude fluctuations intrinsic to a notionally steady noise impede speech perception.

^3^The TE of such a SAM stimulus is given by the equation: *E*(*t*) = 1 + *m*sin(2π*f_m_t*+ϕ), where *m* is the modulation depth, here fixed at 1 (100% modulation), *f_m_* is the modulation frequency, and ϕ is the starting phase of the modulation, which was randomized for each presentation.

^4^Only seven YNH participants completed the SAM detection task.

^5^Results from one YNH participant were not included due to incomplete data.

^6^Levene's test indicated inequality of variance for the two groups. Hence here (and whenever else applicable), corrected degrees of freedom and *t*-values were used.

^7^Due to the unequal size of the two age groups, Cohen's *d* was calculated using the square root of the pooled variance rather than the mean variance (Howell, 2002).

^8^In these correlational analyses, the use of extreme groups (young vs. old) might have increased the power of the significance test but this is considered acceptable for the purpose of proof-of-concept studies (Preacher et al., 2005).
