# Supplementary material for: Age-group differences in speech identification despite matched audiometrically normal hearing: contributions from auditory temporal processing and cognition
Source: Front Aging Neurosci. 2015 Jan 13;6:347. doi: 10.3389/fnagi.2014.00347 (PMC4292733; doi:10.3389/fnagi.2014.00347)
Supplement: Supplementary file 1 [file Presentation1.PDF]

Supplementary Material

Age-group differences in speech identification despite matched audiometrically normal hearing: Contributions from auditory temporal processing and cognition

Christian Füllgrabe[1]*, Brian C. J. Moore[2], Michael A. Stone[3,4]

[1]MRC Institute of Hearing Research, Nottingham, UK
[2]Department of Psychology, University of Cambridge, Cambridge, UK
[3]School of Psychological Sciences, University of Manchester, Manchester, UK
[4]Central Manchester NHS Hospitals Foundation Trust, Manchester, UK

*** Correspondence:** Christian Füllgrabe, MRC Institute of Hearing Research, Science Road, Nottingham, NG7 2RD, UK
christian.fullgrabe@ihr.mrc.ac.uk

Keywords: aging, normal hearing, speech identification, temporal envelope, temporal fine structure, cognition

1. Methodological issues

The consequences of the methodological choices in this study should be pointed out. First, the inclusion only of participants with normal hearing sensitivity resulted in an audiometrically non-representative sample of the older population (see section 1. of the Supplementary Material). Also, our participants were cognitively high-functioning individuals, as indexed by their performance IQ. This co-occurrence might have been caused by a sampling bias (due to our methods and loci of recruitment) or by the fact that hearing *and* cognitive status are both linked to the same underlying variable (such as cardiovascular status, inflammatory factors; Chung et al., 2009; Yamasoba et al., 2013; Nash et al., 2014). Many previous studies of aging also used biased samples, for example because young participants were recruited from the local student body and compared to community dwelling older participants with unknown or difficult-to-quantify educational backgrounds. In the present study, care was taken to match the age groups in terms of years of formal education and age-corrected performance IQ, to allow us to estimate age-group differences in speech perception without any influence of differences in age-normed cognitive functioning. As such, our results provide an estimate of the effects of age on speech-in-noise (SiN) perception for people at the upper limit of auditory and cognitive processing. This knowledge might prove helpful in the fine-tuning of models of speech perception (e.g. the Speech Intelligibility Index; ANSI, 1997), whose predictions could be improved by making "proficiency" a function of age. The results might also be useful in establishing "age-appropriate" targets for speech intelligibility after the provision of hearing devices to older hearing-impaired patients.

A second methodological issue is that "normal hearing" was defined solely as audiometric thresholds within the range that is conventionally considered as normal (here $\leq$ 20 dB HL). Additional measures of peripheral functioning (e.g. auditory filter bandwidth, oto-acoustic emissions, auditory brainstem response) might have been useful in estimating the status of

inner and outer hair cells and auditory neurons. However, the audiogram is still the clinical gold standard for the assessment of hearing health, and in many countries it is the only standard assessment tool. Thus, using audiometric inclusion criteria for participant selection is in line with current clinical practice.

A third methodological issue is that the audiograms of our participants were matched bilaterally only up to 6 kHz. To avoid use of acoustic cues above 6 kHz, our stimuli were lowpass filtered at 6 kHz. Spectral information above 6 kHz can make some contribution to intelligibility, but that contribution is small (ANSI, 1997; Moore et al., 2010). Hence, our choice of 6 kHz as the highest frequency at which audiometric thresholds had to be within the normal range represents a reasonable compromise between experimental feasibility and clinical relevance.

A fourth methodological issue is the need to exclude the possibility that age-group differences in the auditory tasks were due to non-auditory factors. In the present study, to limit procedural and task effects (Robinson and Summerfield, 1996), all participants were given extensive guidance and protracted practice on each auditory task, and their performance was monitored continuously by the experimenter, who intervened in cases of fatigue or inattention. Ideally, it is necessary to demonstrate that young and older participants perform similarly in at least one condition, using the same procedure as used for the experimental conditions. However, such a control condition is rarely used in aging studies. Here, speech identification in quiet was similar for the two groups, indicating that procedural factors were not limiting performance. For supra-threshold auditory tasks (such as our temporal-envelope and temporal-fine-structure sensitivity tasks), it might prove more difficult to show equivalence across age groups, given that aging affects many aspects of auditory processing (Fitzgibbons and Gordon-Salant, 2010). It would be desirable to identify which (if any) auditory processes are age invariant to use them systematically in control conditions in future research; one candidate might be intensity increment detection, which appears to be similar for young normal-hearing (YNH) and older normal-hearing (ONH) listeners (Mazelova et al., 2003; Gallun, 2010).

A fifth methodological issue is connected with sample size. Generally, when correlations are calculated between several dependent variables, large numbers of participants are required to prevent significant correlations from being obtained by chance. Although the numbers in our study were actually larger than in the few other studies using audiometrically matched groups, even larger numbers would be desirable.

Finally, the use of a cross-sectional design introduced the risk of comparing non-equivalent groups differing in characteristics (other than chronological age) that were not assessed in the study but that influence speech perception. In other words, the observed group effects might be the consequence of generation-specific cohort effects and systematic and progressive environmental changes (Baltes et al., 1999; Salthouse, 2010). However, the alternative longitudinal approach (in which each participant acts as his/her own experimental control) comes with its own methodological shortcomings (such as practice effects, selective attrition, and period effects; Salthouse, 2010), and is unsuitable for investigations of people with no hearing deficits, given that, on average, hearing thresholds increase progressively with increasing age.

2. Audiometric screening results for older volunteers with self-reported normal hearing

To put the hearing status of the 21 ONH participants in our study into perspective, Supplementary fig. 1 shows results from an audiometric screening campaign in Nottinghamshire (UK) of volunteers aged 60 years and above that was conducted simultaneously with the study reported in the Original Research article. Recruitment methods were very similar across studies. The advertisement explicitly stated that volunteers needed to have "good hearing" and "no hearing problems". Individual and mean audiograms for 116 older volunteers with self-diagnosed normal hearing (75 females; mean age = 67 years; SD = 6) are shown by the thin and thick black lines, respectively. Coloured lines represent audiograms for volunteers whose audiograms were entirely normal (≤ 20 dB HL between 0.125 and 8 kHz), in one ear (blue) or in both ears (red). For comparison, the mean audiogram ±1 SD for 40 randomly selected young volunteers (< 30 years) with self-diagnosed normal hearing sensitivity, attending screening sessions to qualify for participation in various hearing studies, are shown by the thick white line and light-grey shaded area. Some of these young volunteers had thresholds > 20 dB HL at one or more audiometric frequencies.

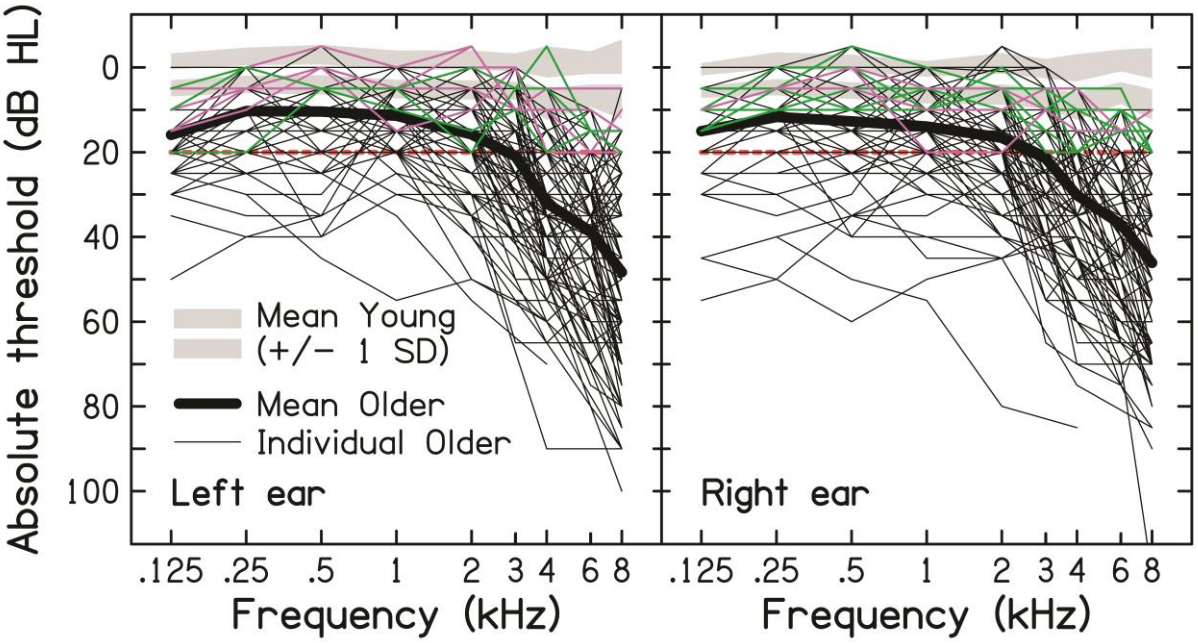

Supplementary figure 1: Results of pure-tone air-conduction audiometry for the left (left panel) and right (right panel) ears of 40 young and 116 older (≥ 60 years) volunteers with self-diagnosed "good" hearing. The thin and thick black lines represent the individual and mean audiograms for the older group, respectively. The thick white lines (and associated light-grey shaded areas) show the mean (±1 SD) audiograms for the young volunteers. The dashed horizontal lines delineate the lower limit of what is conventionally considered as "clinically normal hearing". Note that the *x*- and *y*-axis ranges are different from those in Fig. 1 in the Original Research article. The coloured lines represent audiograms for older volunteers with normal audiometric thresholds (≤ 20 dB HL) between 0.125 and 8 kHz, in one ear (green) or in both ears (pink).

As indicated by the sloping mean audiograms, many older volunteers had a sloping high-frequency hearing loss, despite the fact that all considered their hearing to be good. The mean audiogram corresponds approximately to what has sometimes been labelled as "near-normal hearing" (Pichora-Fuller et al., 1995). The proportion of older volunteers who would be considered as normal hearing, assuming thresholds in both ears ≤ 20 dB HL, for frequencies up to and including 4, 6, and 8 kHz was 0.14, 0.05, and 0.03, respectively. The proportions increased to 0.41, 0.17, and 0.14, respectively, for volunteers with normal hearing in at least one ear. These proportions are roughly consistent with epidemiological data for a UK-population-representative sample reported in the National Study of Hearing (Davis, 1995). The average audiometric thresholds for each ear of our ONH participants follow closely the threshold patterns for the top-fifth percentile of 61- to 70-year-old females, except at 6 kHz, where the present group showed better sensitivity by 10 and 6 dB for the left and right ears, respectively.

These results indicate that many older people who believe that they have good hearing nevertheless have elevated audiometric thresholds, especially at high frequencies.

3. Relationship between cognitive-task performance, age, and speech intelligibility

This section reviews current knowledge of changes in performance during adulthood for each of the cognitive tasks used in this study, and on the association of the latter with speech processing. We also justify the use of each of these tests in our test battery.

3.1. Digit Span (DS) test

Consistent with the notion that short-term memory is relatively unaffected by age (e.g Verhaeghen and Salthouse, 1997; Nilsson, 2003), performance on the Digits Forward (DS-F) test shows no effect of age (Wingfield et al., 1988; Nettelbeck and Rabbitt, 1992; Sliwinski and Buschke, 1999) or relatively small effects (Park et al., 2002), sometimes only apparent above the age of 65 years (Grégoire and Van der Linden, 1997). Since the reordering of the digits in the Digits Backward (DS-B) is assumed to require additional processing in terms of executive control, clinical lore (e.g. The Psychological Corporation, 2002, p. 201-202) suggests that performance on the DS-B test is more affected by age (Strauss et al., 2006). This putative differential sensitivity might explain why DS tests have been repeatedly used in conjunction with SiN identification tasks (see Akeroyd, 2008). However, several studies have failed to find greater age effects for the DS-B than for the DS-F test (Grégoire and Van der Linden, 1997; Myerson et al., 2003; Hester et al., 2004; Wilde et al., 2004). Hence, performance on backward span tasks might require relatively little additional WM processing (Engle et al., 1999). Nevertheless, both DS tests were administered in our study, since Humes et al. (2006) found a positive correlation between composite DS performance and the ability to focus on one talker amongst several.

3.2. Reading Span (RS) test

It is well established that performance on complex working-memory (WM) tests declines with

increasing age (e.g. Fisk and Warr, 1996; Salthouse, 1996; for a meta-analysis, see Bopp and Verhaeghen, 2005), and it has repeatedly been observed that WM, as measured by the RS test, is positively correlated with SiN identification for aided older hearing-impaired participants (Lunner, 2003; Foo et al., 2007; Rudner et al., 2007; Arehart et al., 2013) and unaided young to middle-aged participants with self-reported normal hearing (Ellis and Munro, 2013). WM is also correlated with language comprehension for young listeners (Daneman and Merikle, 1996). Given these robust associations between WM, age, and speech perception, the RS test was included in the cognitive test battery as an indicator of WM capacity.

3.3. Test of Everyday Attention (TEA)

So far, only a few studies have used the TEA to investigate the role of attention in speech identification. Among those, most have focused on specific attentional functions and have restricted their assessment to a sub-set of the TEA (Humes and Coughlin, 2009; Neher et al., 2009; 2011; 2012). An exception is the study of Gatehouse and Akeroyd (2008), in which the entire TEA was administered to a group of older hearing-impaired listeners. Since some of the sub-tests use acoustic stimuli (e.g. in the Elevator Counting with Distraction test, low-frequency tones have to be counted incrementally while ignoring higher-frequency tones), performance might depend somewhat on audiometric status. Robertson et al. (1996) and Gatehouse and Akeroyd (2008) argued that mild-to-moderate hearing loss does not unduly affect test outcomes. However, both empirical (Rabbitt, 1990; Pichora-Fuller et al., 1995; Wingfield et al., 2005) and theoretical work (e.g. Rönnberg et al., 2013) suggest that auditory processing under sub-optimal conditions (e.g. in the presence of hearing loss) leads to higher demands on the cognitive system, thereby reducing the amount of cognitive resources (from a limited pool; Kahneman, 1973) available for the simultaneous execution of other tasks. Therefore, it is possible that hearing loss results in higher processing demands for some of the sub-tests of the TEA. Hence, results from older persons with normal hearing sensitivity would provide unique reference data unbiased by this potentially confounding factor. Here, all eight sub-tests were administered, using version A.

3.4. Trail Making (TM) test

It has been shown that performance on both parts of the TM test declines with increasing age (Kennedy, 1981; Ivnik et al., 1996; Rasmusson et al., 1998), mainly due to declines in speed, not in accuracy (Robins Wahlin et al., 1996). Hence, only time to completion was recorded for each part. We examined the relationship between the raw scores for Part B and a derived measure on one hand and performance on the two speech tasks on the other hand, since executive control functions have been associated with SiN identification (Adank and Janse, 2010; Ellis and Munro, 2013; Woods et al., 2013).

3.5. Block Design (BD) test

Age-related deterioration in performance on the BD test has been shown to be minimal before the age of 55, but accelerates thereafter (Ryan et al., 2000; Rönnlund and Nilsson, 2006). Here, the main purpose of the BD test (combined with the Matrix Reasoning test) was to establish equivalence in age-corrected performance IQ between the young and older participants, but the BD test has also been used, in conjunction with other cognitive tests, to explain individual

differences in speech perception performance (e.g. Jerger et al., 1991; Humes et al., 1994).

3.6. Matrix Reasoning (MR) test

As for the BD test, performance on the MR test has been shown to decline with age (Bugg et al., 2006), and it has been used as part of larger cognitive test batteries (e.g. WAIS-III) to explain individual differences in speech perception performance (e.g. Humes et al., 2007).

4. Grand correlation matrix for the combined group of young and older normal-hearing participants

Supplementary table 1: Pearson product-moment correlation coefficients (top row in each cell) and associated significance levels (two-tailed; bottom row in each cell) for the entire participant group (YNH and ONH participants combined) between audiometric, supra-threshold auditory processing, cognitive, and speech measures: **(1) Age** (in years); **(2) PTA**: bilateral pure-tone average for frequencies 0.125-6 kHz (in dB HL); **(3) Consonants**: Composite consonant identification scores (in RAUs) for the unmodulated, 5-Hz amplitude-modulated, and 80-Hz amplitude-modulated background noises; **(4) MMR**: Composite modulation masking release (in RAUs) for the 5-Hz and 80-Hz amplitude-modulated background noises at signal-to-noise ratios (SNRs) of −6, −10, and −14 dB; **(5) Sentences**: Composite sentence identification scores (in RAUs) in the presence of co-located and separate interfering talkers; **(6) TE**: Composite sensitivity to temporal-envelope information in $20\log_{10}(m)$; **(7) Monaural TFS**: Composite sensitivity to temporal-fine-structure information (in d′) on the monaural task using center frequencies of 1 and 2 kHz; **(8) Binaural TFS**: Composite sensitivity to temporal-fine-structure information (in d′) on the binaural task using the two frequencies of 0.5 and 0.75 kHz; **(9) TFS**: Composite sensitivity to temporal-fine-structure information (in d′) on the monaural and binaural tasks; **(10) DS-F**: Performance (in *z* scores) on the Digits Forward test; **(11) DS-B**: Performance (in *z* scores) on the Digits Backward test; **(12) RS**: Performance (in *z* scores) on the Reading Span test; **(13) TEA**: Performance (in *z* scores) on the Test of Everyday Attention; **(14) MS**: Performance (in *z* scores) on the TEA sub-test *Map Search*; **(15) TS**: Performance (in *z* scores) on the TEA sub-test *Telephone Search*; **(16) ECD**: Performance (in *z* scores) on the TEA sub-test *Elevator Counting with Distraction*; **(17) ECR**: Performance (in *z* scores) on the TEA sub-test *Elevator Counting with Reversal*; **(18) EC**: Performance (in *z* scores) on the TEA sub-test *Elevator Counting*; **(19) Lottery**: Performance (in *z* scores) on the TEA sub-test *Lottery*; **(20) TSC**: Performance (in *z* scores) on the TEA sub-test *Telephone Search wile Counting*; **(21) VE**: Performance (in *z* scores) on the TEA sub-test *Visual Elevator*; **(22) TM B**: Performance (in *z* scores) on the Trail Making test, Part B; **(23) TM [(B−A)/A]**: Derived measure (in *z* scores) of the Trail Making test, [(Part B−Part A)/Part A]; **(24) BD**: Performance (in *z* scores) on the Block Design test; **(25) MR**: Performance (in *z* scores) on the Matrix Reasoning test; **(26) Cognition**: Composite performance (in *z* scores) on the six cognitive tests [(10), (11), (12), (22), (24), (25)] and eight TEA sub-tests [(14)-(21)]. Grey and black values indicate non-significant and significant group differences (at $p \leq 0.05$), respectively.

	**(2)**	**(3)**	**(4)**	**(5)**	**(6)**	**(7)**	**(8)**	**(9)**	**(10)**	**(11)**	**(12)**	**(13)**	**(14)**	**(15)**	**(16)**	**(17)**	**(18)**	**(19)**	**(20)**	**(21)**	**(22)**	**(23)**	**(24)**	**(25)**	**(26)**
Age **(1)**	.20<br>.284	-.71<br>.001	.08<br>.668	-.70<br>.001	-.38<br>.046	-.67<br>.001	-.59<br>.001	-.68<br>.001	-.15<br>.426	-.13<br>.494	-.56<br>.001	-.49<br>.006	-.65<br>.001	-.47<br>.009	.23<br>.215	-.42<br>.021	.03<br>.870	-.10<br>.595	-.08<br>.694	.47<br>.008	-.59<br>.001	.07<br>.727	-.68<br>.001	-.70<br>.001	-.59<br>.001
PTA **(2)**		-.11<br>.571	.06<br>.766	-.34<br>.065	-.08<br>.677	-.27<br>.158	-.30<br>.114	-.32<br>.086	-.54<br>.002	-.29<br>.115	-.31<br>.105	-.41<br>.024	-.34<br>.069	-.01<br>.940	-.07<br>.703	-.14<br>.459	-.12<br>.485	-.11<br>.562	-.63<br>.001	-.18<br>.346	-.24<br>.212	-.04<br>.840	-.24<br>.195	-.32<br>.087	-.44<br>.018
Consonants **(3)**			-.19<br>.319	.77<br>.001	-.56<br>.002	.64<br>.001	.72<br>.001	.78<br>.001	.45<br>.013	.27<br>.156	.46<br>.011	.64<br>.001	.67<br>.001	.59<br>.001	.05<br>.805	.59<br>.001	-.08<br>.664	.28<br>.137	.02<br>.906	.40<br>.030	.64<br>.001	-.04<br>.833	.82<br>.001	.59<br>.001	.70<br>.001
MMR **(4)**				-.13<br>.483	.28<br>.148	-.07<br>.706	-.27<br>.153	-.23<br>.224	-.07<br>.719	-.11<br>.577	-.16<br>.419	-.36<br>.048	-.27<br>.149	-.30<br>.106	-.20<br>.290	-.16<br>.398	-.32<br>.085	.19<br>.307	-.07<br>.703	-.32<br>.085	-.21<br>.286	-.04<br>.842	-.11<br>.580	-.16<br>.391	-.20<br>.303
Sentences **(5)**					-.45<br>.017	.76<br>.001	.70<br>.001	.81<br>.001	.58<br>.001	.42<br>.021	.54<br>.003	.59<br>.001	.64<br>.001	.326<br>.079	.07<br>.718	.54<br>.002	.04<br>.832	.04<br>.821	.26<br>.164	.42<br>.021	.76<br>.001	.18<br>.365	.76<br>.001	.60<br>.001	.81<br>.001
TE **(6)**						-.41<br>.031	-.70<br>.001	-.68<br>.001	-.20<br>.317	-.20<br>.307	-.28<br>.153	-.54<br>.003	-.58<br>.001	-.53<br>.004	-.11<br>.575	-.47<br>.012	-.09<br>.634	.02<br>.941	.02<br>.916	-.34<br>.073	-.41<br>.030	.15<br>.461	-.45<br>.016	-.29<br>.139	-.38<br>.046
Monaural TFS **(7)**							.54<br>.002	.77<br>.001	.35<br>.064	.33<br>.076	.32<br>.095	.45<br>.013	.61<br>.001	.51<br>.005	.00<br>.988	.42<br>.022	-.21<br>.278	.07<br>.714	-.02<br>.916	.41<br>.028	.58<br>.001	.01<br>.946	.68<br>.001	.64<br>.001	.61<br>.001
Binaural TFS **(8)**								.95<br>.001	.44<br>.017	.34<br>.073	.39<br>.037	.69<br>.001	.70<br>.001	.40<br>.033	.10<br>.622	.65<br>.001	.02<br>.915	.11<br>.576	.21<br>.270	.54<br>.003	.70<br>.001	.10<br>.618	.72<br>.001	.55<br>.002	.69<br>.001
TFS **(9)**									.46<br>.012	.38<br>.044	.41<br>.028	.69<br>.001	.75<br>.001	.48<br>.008	.07<br>.704	.64<br>.001	-.06<br>.762	.11<br>.578	.15<br>.426	.55<br>.002	.73<br>.001	.08<br>.687	.79<br>.001	.64<br>.001	.74<br>.001
DS-F **(10)**										.62<br>.001	.27<br>.155	.67<br>.001	.36<br>.051	.20<br>.288	.36<br>.051	.54<br>.002	.19<br>.307	.07<br>.722	.51<br>.004	.39<br>.032	.44<br>.016	-.04<br>.841	.46<br>.011	.47<br>.010	.70<br>.001
DS-B **(11)**											.17<br>.384	.43<br>.018	.13<br>.494	.00<br>.993	.40<br>.030	.42<br>.021	-.00<br>.983	-.06<br>.768	.43<br>.019	.37<br>.044	.47<br>.010	.09<br>.640	.39<br>.034	.39<br>.031	.66<br>.001
RS **(12)**												.31<br>.105	.54<br>.003	.15<br>.438	-.13<br>.489	.31<br>.103	-.11<br>.582	.11<br>.582	.02<br>.931	.34<br>.075	.53<br>.003	.25<br>.195	.66<br>.001	.54<br>.003	.67<br>.001
TEA **(13)**													.66<br>.001	.51<br>.004	.45<br>.013	.70<br>.001	.33<br>.071	.18<br>.341	.48<br>.007	.63<br>.001	.57<br>.001	-.04<br>.852	.57<br>.001	.57<br>.001	.70<br>.001
MS **(14)**														.55<br>.002	-.15<br>.417	.40<br>.028	-.04<br>.830	.29<br>.118	.18<br>.331	.37<br>.045	.55<br>.002	.02<br>.912	.72<br>.001	.60<br>.001	.63<br>.001
TS **(15)**															0.6<br>.767	.34<br>.067	-.12<br>.518	.00<br>.984	-.20<br>.283	.37<br>.045	.36<br>.056	-.35<br>.062	.52<br>.004	-.37<br>.043	.29<br>.125
ECD **(16)**																.48<br>.008	.10<br>.619	-.10<br>.613	.16<br>.393	.22<br>.245	.09<br>.639	.04<br>.840	.00<br>.991	-.08<br>.688	.18<br>.364
ECR **(17)**																	-.12<br>.546	-.24<br>.202	.14<br>.454	.77<br>.001	.56<br>.002	.15<br>.446	.59<br>.001	.52<br>.004	.67<br>.001
EC **(18)**																		.08<br>.695	.42<br>.020	.00<br>1	-.03<br>.896	-.11<br>.586	-.33<br>.072	-.06<br>.774	-.05<br>.785
Lottery **(19)**																			.06<br>.742	-.38<br>.039	-.07<br>.723	-.12<br>.546	.18<br>.347	.05<br>.806	.05<br>.802
TSC **(20)**																				.12<br>.520	.15<br>.429	.05<br>.799	.08<br>.676	.17<br>.372	.32<br>.091
VE **(21)**																					.61<br>.001	.17<br>.377	.48<br>.008	.68<br>.001	.65<br>.001
TM B **(22)**																						.46<br>.013	.77<br>.001	.52<br>.004	.87<br>.001
TM (B-A)/A **(23)**																							.14<br>.470	-.11<br>.585	.34<br>.070
BD **(24)**																								.66<br>.001	.85<br>.001
MR **(25)**																									.73<br>.001
Cognition **(26)**																									

5. Raw scores and statistical results for cognitive measures

Supplementary table 2: (**A**) Group-mean performance for the YNH and ONH participants, and results from independent-samples *t* tests (degrees of freedom, *df*; *t* value, *t*; *p* value, *p*) for z-score-transformed performance on each of the seven cognitive tests and for the derived measure for the TM test. The maximum score or measurement unit is given in parentheses next to the test name. The sub-tests of the TEA used different measurement units; hence, the only global TEA score that could be computed was the average of the *z* scores for each of its sub-tests. Grey and black values indicate non-significant and significant group differences (at $p \leq 0.05$), respectively. Values in boldface denote significant results after applying a Holm-Bonferroni correction. (**B**) Group-mean performance and associated statistical results for each of the eight sub-tests of the TEA. Otherwise as (A).

**(A)** *Cognitive measures (maximum score or unit)*	Performance		Statistical results		
	YNH	ONH	*df*	*t*	*p*
Digits Forward (/88)	51.0	48.4	25.324	−0.749	0.461
Digits Backward (/70)	35.2	32.4	28	−0.634	0.531
Reading Span (/54)	**34.9**	**28.3**	**27**	**−3.989**	**< 0.001**
Test of Everyday Attention	/	/	**28**	**−2.715**	**0.011**
Trail Making B (s)	**35.2**	**55.7**	**23.195**	**−4.954**	**< 0.001**
Trail Making [(B−A)/A]	0.70	0.69	27	−0.046	0.964
Block Design (/71)	**62.7**	**42.7**	**21.072**	**−6.258**	**< 0.001**
Matrix Reasoning (/35)	**32.8**	**28.7**	**28**	**−4.702**	**< 0.001**

**(B)** *TEA sub-tests (maximum score or unit)*	Performance		Statistical results		
	YNH	ONH	*df*	*t*	*p*
Map Search (/80)	**75.4**	**59.7**	**27.568**	**−6.361**	**< 0.001**
Telephone Search (s)	2.61	3.32	28	−2.628	0.014
Elevator Counting with Distraction (/10)	7.78	8.95	28	1.719	0.097
Elevator Counting with Reversal (/10)	**9.44**	**7.33**	**23.243**	**−3.447**	**0.002**
Elevator Counting (/7)	6.89	6.95	28	0.621	0.539
Lottery (/10)	9.78	9.62	28	−0.650	0.521
Telephone Search while Counting (s)	0.45	0.45	28	−0.039	0.969
Visual Elevator (s)	3.38	4.38	28	−2.451	0.021

6. Discrepancy between measured and self-assessed hearing difficulties

Somewhat unexpectedly, the measured deficits in speech identification for the ONH participants were not reflected in their subjective evaluation of their hearing (dis)abilities[1]. Possible explanations for this include: (1) the ONH group's (vicarious) experience of serious age-related health issues or conditions of hardship may make them perceive speech-perception difficulties as less serious (Cassileth et al., 1984; Deeg et al., 1996); (2) the lifestyle of the ONH group may place less demands on hearing (e.g. they may chose a more restricted set of activities and communication environments), making them experience speech-perception difficulties less frequently; (3) the ONH group may behave as "health optimists" (Idler, 1993) and may evaluate their hearing abilities relative to those of their age peers, who, on average, suffer from hearing loss (see section 1.1. in the Supplementary Material), and consequently have greater speech-perception deficits; (4) cognitive processing of the items in the research instrument may differ for young and older participants (Schwartz, 1998).

Echoing earlier claims (Wiley et al., 2000), our results suggest a need for caution in interpreting the results of self-report inventories when studying hearing disability and handicap across age groups. Whatever the reason for the lack of difference in subjective evaluation across age groups, our finding is at odds with the report of Banh et al. (2012) of significantly lower scores on the Speech, Spatial, and Qualities of hearing scale (SSQ) for older than for young listeners, all with "clinically normal hearing". However, Banh et al. (2012) used less stringent audiometric inclusion criteria (thresholds ≤ 25 dB HL only up to 3 kHz and averaged across ears). Also, as pointed out by Gatehouse and Noble (2004), responses on the SSQ are probably partly a function of the respondent's cognitive abilities. Hence, in contrast to the present study in which age groups were matched in terms of average audibility and performance IQ, Banh et al. (2012) might have observed an age effect on SSQ responses that was mediated by age differences in hearing sensitivity at high frequencies (> 3 kHz) and/or in cognitive abilities.

7. Confounding age effect in a study of hearing loss

In a recent study (Moore et al., 2010), we aimed to study the benefit of extending the bandwidth of hearing aids for speech-in-speech identification. The same speech and masker material and spatial conditions as in the present study (see section 2.3.2. of the Original Research) were used, with the exception that the target-and-masker mixture was lowpass filtered at 10 kHz. Eight normal-hearing participants and 16 participants with mild-to-moderate hearing loss were tested. The hearing-impaired group was substantially older (mean age = 71 years; range = 46-82) than the normal-hearing group (mean age = 23 years; range = 19-33).

In the reference condition, all participants were tested unaided. The hearing-impaired group performed markedly more poorly than the normal-hearing group, an effect that was implicitly

[1] However, when asked during the initial clinical interview to comment on personally experienced difficult listening situations, the ONH participants volunteered examples (e.g. a crowded restaurant) more often than the YNH participants. Such a discrepancy between responses to a standardized questionnaire and self-reported communication difficulties was also reported by Dalton et al. (2003).

ascribed to the difference in audiograms. However, age-related differences in central auditory processing and cognitive abilities between the two groups could also have contributed to the differences in speech intelligibility. To test this hypothesis, here we compare the mean identification scores in the presence of co-located and separate two-talker interference at different SNRs for the older hearing-impaired (OHI) participants from Moore et al. (2010) and the ONH participants from the present study; the two groups had similar mean ages (71 and 67 years, respectively). Supplementary fig. 2 indicates that, on average, the OHI participants had worse hearing sensitivity than the ONH participants at all audiometric frequencies, especially for frequencies above 1 kHz.

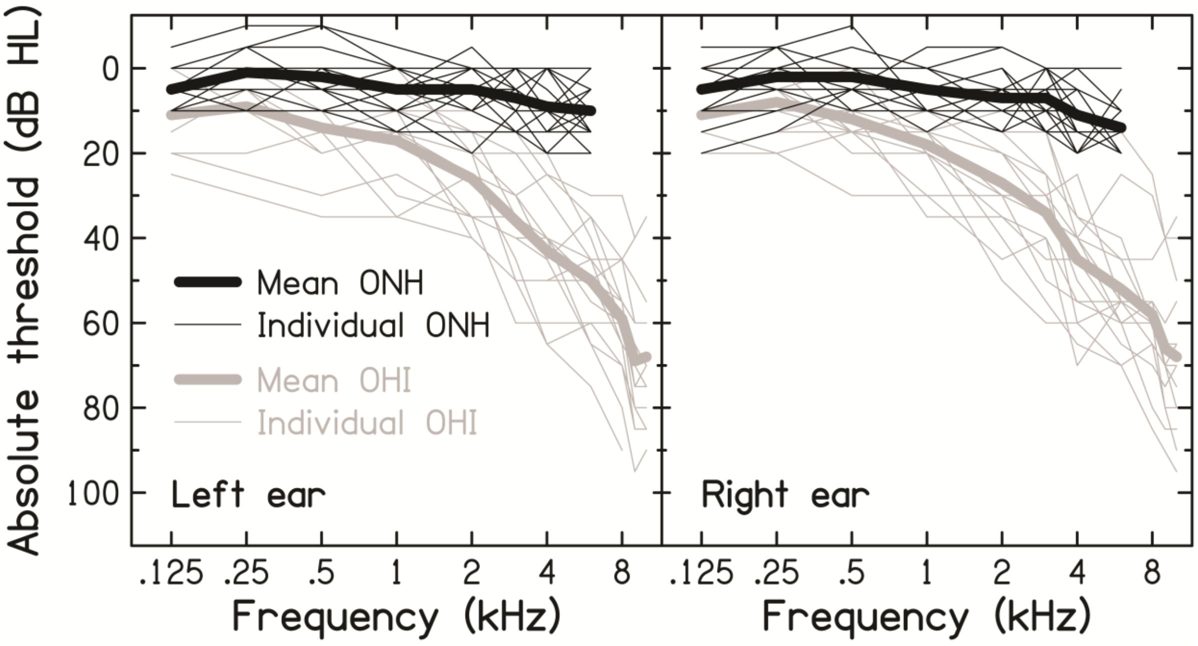

Supplementary figure 2: Individual and mean pure-tone air-conduction audiometric thresholds for the 21 ONH participants (black lines) from the present study and 16 OHI participants (grey lines) from Moore et al. (2010). Note that the highest audiometric frequency assessed in the two studies was 6 and 10 kHz, respectively.

Even though only partially overlapping SNRs and different bandwidths were used in the two studies, the results, shown in Supplementary fig. 3, are roughly comparable. Surprisingly, the (unaided) OHI participants, despite their reduction in audibility, showed similar performance in the co-located condition to the ONH participants, indicating that age, rather than hearing loss, determines speech-in-speech intelligibility in conditions where spatial release from masking (SMR) does not occur. In contrast, in the separate condition, the OHI participants performed much worse than the ONH participants (*i.e.*, the former showed substantially less SMR). This suggests that hearing loss was the main contributor to poor speech-in-speech intelligibility in this condition. These results illustrate the possibility of an age confound in studies investigating the perceptual consequences of hearing loss for speech perception and other auditory processing abilities.

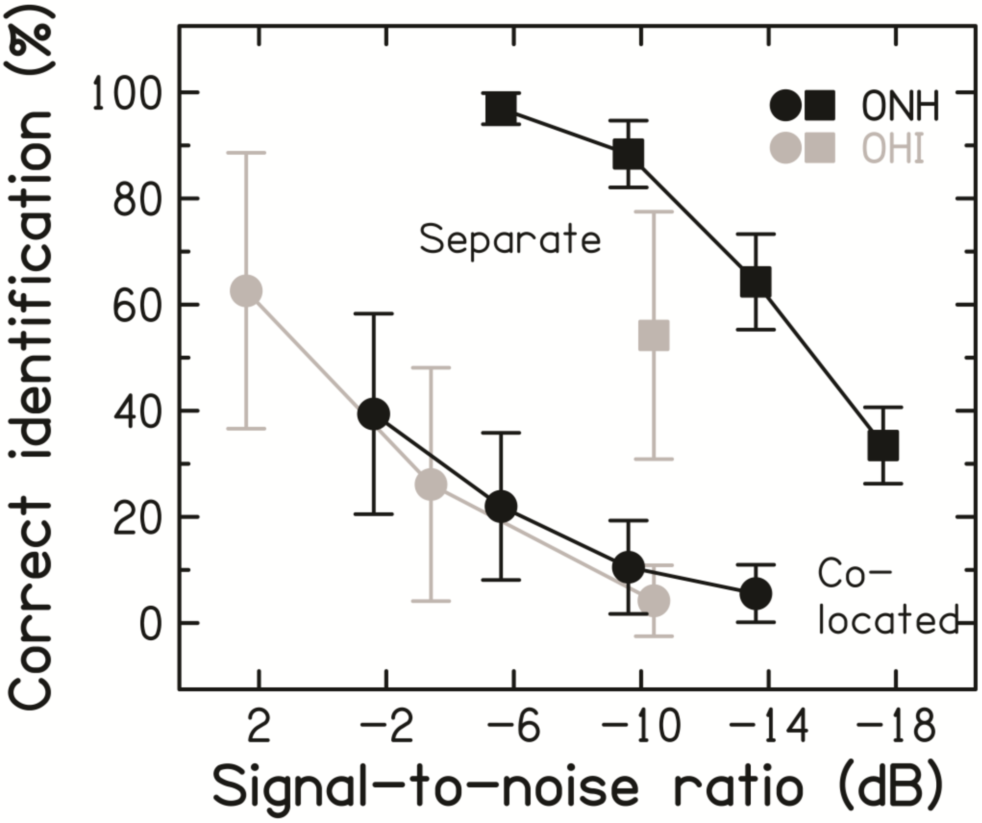

Supplementary figure 3: Average identification of speech in two-talker interference for the ONH (black symbols) and OHI (grey symbols) participants. Scores are given as a function of SNR for the co-located (circles) and the separate conditions (squares). Error bars represent ±1 SD.

8. References

Adank, P., and Janse, E. (2010). Comprehension of a novel accent by young and older listeners. *Psychol. Aging* 25**,** 736-740. doi: 10.1037/a0020054.

Akeroyd, M.A. (2008). Are individual differences in speech reception related to individual differences in cognitive ability? A survey of twenty experimental studies with normal and hearing-impaired adults. *Int. J. Audiol.* 47 Suppl 2**,** S53-71. doi: 10.1080/14992020802301142.

ANSI (1997). *S3.5-1997. Methods for the Calculation of the Speech Intelligibility Index.* New York: American National Standards Institute.

Arehart, K.H., Souza, P., Baca, R., and Kates, J.M. (2013). Working memory, age, and hearing loss: susceptibility to hearing aid distortion. *Ear Hear.* 34**,** 251-260. doi: 10.1097/AUD.0b013e318271aa5e.

Baltes, P.B., Staudinger, U.M., and Lindenberger, U. (1999). Lifespan psychology: theory and application to intellectual functioning. *Annu. Rev. Psychol.* 50**,** 471-507. doi: 10.1146/annurev.psych.50.1.471.

Banh, J., Singh, G., and Pichora-Fuller, M.K. (2012). Age affects responses on the Speech, Spatial, and Qualities of Hearing Scale (SSQ) by adults with minimal audiometric loss. *J. Am. Acad. Audiol.* 23**,** 81-91.

Bopp, K.L., and Verhaeghen, P. (2005). Aging and verbal memory span: a meta-analysis. *J. Gerontol. B Psychol. Sci. Soc. Sci.* 60**,** P223-233.

Bugg, J.M., Zook, N.A., Delosh, E.L., Davalos, D.B., and Davis, H.P. (2006). Age differences in fluid intelligence: contributions of general slowing and frontal decline. *Brain Cogn.* 62**,** 9-16. doi: 10.1016/j.bandc.2006.02.006.

Cassileth, B.R., Lusk, E.J., Strouse, T.B., Miller, D.S., Brown, L.L., Cross, P.A., and Tenaglia, A.N. (1984). Psychosocial status in chronic illness - a comparative-analysis of 6 diagnostic groups. *N. Engl. J. Med.* 311**,** 506-511. doi: 10.1056/Nejm198408233110805.

Chung, H.Y., Cesari, M., Anton, S., Marzetti, E., Giovannini, S., Seo, A.Y., Carter, C., Yu, B.P., and Leeuwenburgh, C. (2009). Molecular inflammation: underpinnings of aging and age-related diseases. *Ageing Res. Rev.* 8**,** 18-30. doi: 10.1016/j.arr.2008.07.002.

Dalton, D.S., Cruickshanks, K.J., Klein, B.E., Klein, R., Wiley, T.L., and Nondahl, D.M. (2003). The impact of hearing loss on quality of life in older adults. *Gerontologist* 43**,** 661-668.

Daneman, M., and Merikle, P.M. (1996). Working memory and language comprehension: A meta-analysis. *Psychon. Bull. Rev.* 3**,** 422-433. doi: 10.3758/BF03214546.

Davis, A. (1995). *Hearing in Adults.* London: Whurr.

Deeg, D.J.H., Kardaun, J.W.P.F., and Forzard, J.L. (1996). "Health, behaviour, and aging," in *Handbook of the Psychology on Aging,* eds. J.E. Birren & K.W. Schaie. (New York: Academic), 129-149.

Ellis, R.J., and Munro, K.J. (2013). Does cognitive function predict frequency compressed speech recognition in listeners with normal hearing and normal cognition? *Int. J. Audiol.* 52**,** 14-22. doi: 10.3109/14992027.2012.721013.

Engle, R.W., Tuholski, S.W., Laughlin, J.E., and Conway, A.R. (1999). Working memory, short-term memory, and general fluid intelligence: a latent-variable approach. *J. Exp. Psychol. Gen.* 128**,** 309-331.

Fisk, J.E., and Warr, P. (1996). Age and working memory: the role of perceptual speed, the central executive, and the phonological loop. *Psychol. Aging* 11**,** 316-323.

Fitzgibbons, P.J., and Gordon-Salant, S. (2010). "Behavioural studies with aging humans: Hearing sensitivity and psychoacoustics," in *The Aging Auditory System,* eds. S. Gordon-Salant, R.D. Frisina, A.N. Popper & R.R. Fay. (Heidelberg: Springer), 111-134.

Foo, C., Rudner, M., Rönnberg, J., and Lunner, T. (2007). Recognition of speech in noise with new hearing instrument compression release settings requires explicit cognitive storage and processing capacity. *J. Am. Acad. Audiol.* 18**,** 618-631.

Gallun, F.J. (2010). Judgments of intensity for brief sequences. *J. Acoust. Soc. Am.* 127**,** EL166-171. doi: 10.1121/1.3359815.

Gatehouse, S., and Akeroyd, M.A. (2008). The effects of cueing temporal and spatial attention on word recognition in a complex listening task in hearing-impaired listeners. *Trends Amplif.* 12**,** 145-161. doi: 10.1177/1084713808317395.

Gatehouse, S., and Noble, W. (2004). The Speech, Spatial and Qualities of Hearing Scale (SSQ). *Int. J. Audiol.* 43**,** 85-99. doi: 10.1080/14992020400050014.

Grégoire, J., and Van Der Linden, M. (1997). Effect of age on forward and backward digit spans. *Aging Neuropsychol. Cogn.* 4**,** 140-149.

Hester, R.L., Kinsella, G.J., and Ong, B. (2004). Effect of age on forward and backward span tasks. *J. Int. Neuropsychol. Soc.* 10**,** 475-481. doi: 10.1017/S1355617704104037.

Humes, L.E., Burk, M.H., Coughlin, M.P., Busey, T.A., and Strauser, L.E. (2007). Auditory speech recognition and visual text recognition in younger and older adults: similarities

and differences between modalities and the effects of presentation rate. *J. Speech Lang. Hear. Res.* 50**,** 283-303. doi: 10.1044/1092-4388(2007/021).

Humes, L.E., and Coughlin, M.P. (2009). Aided speech-identification performance in single-talker competition by older adults with impaired hearing. *Scand. J. Psychol.* 50**,** 485-494. doi: 10.1111/j.1467-9450.2009.00740.x.

Humes, L.E., Lee, J.H., and Coughlin, M.P. (2006). Auditory measures of selective and divided attention in young and older adults using single-talker competition. *J. Acoust. Soc. Am.* 120**,** 2926-2937.

Humes, L.E., Watson, B.U., Christensen, L.A., Cokely, C.G., Halling, D.C., and Lee, L. (1994). Factors associated with individual differences in clinical measures of speech recognition among the elderly. *J. Speech Hear. Res.* 37**,** 465-474.

Idler, E.L. (1993). Age differences in self-assessments of health: age changes, cohort differences, or survivorship? *J. Gerontol.* 48**,** S289-300.

Ivnik, R.J., Malec, J.F., Smith, G.E., Tangalos, E.G., and Petersen, R.C. (1996). Neuropsychological tests' norms above age 55: COWAT, BNT, MAE token, WRAT-R reading, AMNART, STROOP, TMT, and JLO. *Clin. Neuropsychol.* 10**,** 262-278.

Jerger, J., Jerger, S., and Pirozzolo, F. (1991). Correlational analysis of speech audiometric scores, hearing loss, age, and cognitive abilities in the elderly. *Ear Hear.* 12**,** 103-109.

Kahneman, D. (1973). *Attention and Effort.* New Jersey: Prentice-Hall.

Kennedy, K.J. (1981). Age effects on Trail Making Test performance. *Percept. Motor Skills* 52**,** 671-675. doi: 10.2466/pms.1981.52.2.671.

Lunner, T. (2003). Cognitive function in relation to hearing aid use. *Int. J. Audiol.* 42 Suppl 1**,** S49-58.

Mazelova, J., Popelar, J., and Syka, J. (2003). Auditory function in presbycusis: peripheral vs. central changes. *Exp. Gerontol.* 38**,** 87-94.

Moore, B.C.J., Füllgrabe, C., and Stone, M.A. (2010). Effect of spatial separation, extended bandwidth, and compression speed on intelligibility in a competing-speech task. *J. Acoust. Soc. Am.* 128**,** 360-371. doi: 10.1121/1.3436533.

Myerson, J., Emery, L., White, D.A., and Hale, S. (2003). Effects of age, domain, and processing demands on memory span: Evidence for differential decline. *Aging Neuropsychol. Cogn.* 10**,** 20-27.

Nash, S.D., Cruickshanks, K.J., Zhan, W., Tsai, M.Y., Klein, R., Chappell, R., Nieto, F.J., Klein, B.E., Schubert, C.R., Dalton, D.S., and Tweed, T.S. (2014). Long-term assessment of systemic inflammation and the cumulative incidence of age-related hearing impairment in the epidemiology of hearing loss study. *J. Gerontol. A Biol. Sci. Med. Sci.* 69**,** 207-214. doi: 10.1093/gerona/glt075.

Neher, T., Behrens, T., Carlile, S., Jin, C., Kragelund, L., Petersen, A.S., and Schaik, A. (2009). Benefit from spatial separation of multiple talkers in bilateral hearing-aid users: Effects of hearing loss, age, and cognition. *Int. J. Audiol.* 48**,** 758-774. doi: 10.3109/14992020903079332.

Neher, T., Laugesen, S., Jensen, N.S., and Kragelund, L. (2011). Can basic auditory and cognitive measures predict hearing-impaired listeners' localization and spatial speech recognition abilities? *J. Acoust. Soc. Am.* 130**,** 1542-1558. doi: 10.1121/1.3608122.

Neher, T., Lunner, T., Hopkins, K., and Moore, B.C.J. (2012). Binaural temporal fine structure sensitivity, cognitive function, and spatial speech recognition of hearing-impaired listeners (L). *J. Acoust. Soc. Am.* 131**,** 2561-2564. doi: 10.1121/1.3689850.

Nettelbeck, T., and Rabbitt, P. (1992). Aging, cognitive performance, and mental speed. *Intelligence* 16**,** 189-205.

Nilsson, L.-G. (2003). Memory function in normal aging. *Acta Neurol. Scand. Suppl.* 179**,** 7-13.

Park, D.C., Lautenschlager, G., Hedden, T., Davidson, N.S., Smith, A.D., and Smith, P.K. (2002). Models of visuospatial and verbal memory across the adult life span. *Psychol. Aging* 17**,** 299-320.
Pichora-Fuller, M.K., Schneider, B.A., and Daneman, M. (1995). How young and old adults listen to and remember speech in noise. *J. Acoust. Soc. Am.* 97**,** 593-608.
Rabbitt, P. (1990). Mild hearing loss can cause apparent memory failures which increase with age and reduce with IQ. *Acta Otolaryngol. Suppl.* 476**,** 167-175.
Rasmusson, X.D., Zonderman, A.B., Kawas, C., and Resnick, S.M. (1998). Effects of age and dementia on the Trail Making Test. *Clin. Neuropsychol.* 12**,** 169-178.
Robertson, I.H., Ward, T., Ridgeway, V., and Nimmo-Smith, I. (1996). The structure of normal human attention: The Test of Everyday Attention. *J. Int. Neuropsychol. Soc.* 2**,** 525-534.
Robins Wahlin, T.B., Bäckman, L., Wahlin, Å., and Winblad, B. (1996). Trail Making Test performance in a community-based sample of healthy very old adults: effects of age on completion time, but not on accuracy. *Arch. Gerontol. Geriatr.* 22**,** 87-102.
Robinson, K., and Summerfield, A.Q. (1996). Adult auditory learning and training. *Ear Hear.* 17**,** S51-S65. doi: 10.1097/00003446-199617031-00006.
Rönnberg, J., Lunner, T., Zekveld, A., Sörqvist, P., Danielsson, H., Lyxell, B., Dahlstrom, O., Signoret, C., Stenfelt, S., Pichora-Fuller, M.K., and Rudner, M. (2013). The Ease of Language Understanding (ELU) model: theoretical, empirical, and clinical advances. *Front. Syst. Neurosci.* 7**,** 31. doi: 10.3389/fnsys.2013.00031.
Rönnlund, M., and Nilsson, L.-G. (2006). Adult life-span patterns in WAIS-R Block Design performance: Cross-sectional versus longitudinal age gradients and relations to demographic factors. *Intelligence* 34**,** 63-78.
Rudner, M., Foo, C., Rönnberg, J., and Lunner, T. (2007). Phonological mismatch makes aided speech recognition in noise cognitively taxing. *Ear Hear.* 28**,** 879-892. doi: 10.1097/AUD.0b013e3181576c9c.
Ryan, J.J., Sattler, J.M., and Lopez, S.J. (2000). Age effects on Wechsler Adult Intelligence Scale-III subtests. *Arch. Clin. Neuropsychol.* 15**,** 311-317.
Salthouse, T.A. (1996). The processing-speed theory of adult age differences in cognition. *Psychol. Rev.* 103**,** 403-428.
Salthouse, T.A. (2010). *Major Issues in Cognitive Aging.* New York: Oxford University Press.
Schwartz, N. (1998). "Self-reports of behaviors and opinions: Cognitive and communicative processes," in *Cognition, Aging, and Self-Reports,* eds. N. Schwartz, D.C. Park, B. Knäuper & S. Sudman. (Hove, UK: Psychology Press), 17-43.
Sliwinski, M., and Buschke, H. (1999). Cross-sectional and longitudinal relationships among age, cognition, and processing speed. *Psychol. Aging* 14**,** 18-33. doi: 10.1037//0882-7974.14.1.18.
Strauss, E., Sherman, E.M.S., and Spreen, O. (eds.). (2006). *A Compendium of Neuropsychological Tests.* New York: Oxford University Press.
Verhaeghen, P., and Salthouse, T.A. (1997). Meta-analyses of age-cognition relations in adulthood: estimates of linear and nonlinear age effects and structural models. *Psych. Bull.* 122**,** 231-249.
Wilde, N.J., Strauss, E., and Tulsky, D.S. (2004). Memory span on the Wechsler scales. *J. Clin. Exp. Neuropsychol.* 26**,** 539-549. doi: 10.1080/13803390490496605.
Wiley, T.L., Cruickshanks, K.J., Nondahl, D.M., and Tweed, T.S. (2000). Self-reported hearing handicap and audiometric measures in older adults. *J. Am. Acad. Audiol.* 11**,** 67-75.
Wingfield, A., Stine, E.A.L., Lahar, C.J., and Aberdeen, J.S. (1988). Does the capacity of working memory change with age? *Exp. Aging Res.* 14**,** 103-107. doi: 10.1080/03610738808259731.

Wingfield, A., Tun, P.A., and McCoy, S.L. (2005). Hearing loss in older adulthood - What it is and how it interacts with cognitive performance. *Curr. Dir. Psychol. Sci.* 14**,** 144-148. doi: DOI 10.1111/j.0963-7214.2005.00356.x.
Woods, W.S., Kalluri, S., Pentony, S., and Nooraei, N. (2013). Predicting the effect of hearing loss and audibility on amplified speech reception in a multi-talker listening scenario. *J. Acoust. Soc. Am.* 133**,** 4268-4278. doi: 10.1121/1.4803859.
Yamasoba, T., Lin, F.R., Someya, S., Kashio, A., Sakamoto, T., and Kondo, K. (2013). Current concepts in age-related hearing loss: epidemiology and mechanistic pathways. *Hear. Res.* 303**,** 30-38. doi: 10.1016/j.heares.2013.01.021.
